# A common developmental program can produce diverse leaf shapes

**DOI:** 10.1111/nph.14449

**Published:** 2017-03-01

**Authors:** Adam Runions, Miltos Tsiantis, Przemyslaw Prusinkiewicz

**Affiliations:** ^1^ University of Calgary 2500 University Dr NW Calgary Alberta T2N 1N4 Canada; ^2^ Max Planck Institute for Plant Breeding Research Carl‐von‐Linné‐Weg 10 Köln 50829 Germany

**Keywords:** blastozone, computational model, directional growth, Hofmeister's rule, leaf development, PIN convergence point, shape diversity, vascular system

## Abstract

- Eudicot leaves have astoundingly diverse shapes. The central problem addressed in this paper is the developmental origin of this diversity.
- To investigate this problem, we propose a computational model of leaf development that generalizes the largely conserved molecular program for the reference plants *Arabidopsis thaliana*,* Cardamine hirsuta* and *Solanum lycopersicum*. The model characterizes leaf development as a product of three interwoven processes: the patterning of serrations, lobes and/or leaflets on the leaf margin; the patterning of the vascular system; and the growth of the leaf blade spanning the main veins. The veins play a significant morphogenetic role as a local determinant of growth directions.
- We show that small variations of this model can produce diverse leaf shapes, from simple to lobed to compound.
- It is thus plausible that diverse shapes of eudicot leaves result from small variations of a common developmental program.

## Abstract 2

See also the Commentary on this article by Thomas & Ougham, **216**: 337–338.

## Introduction

**Figure 1:**
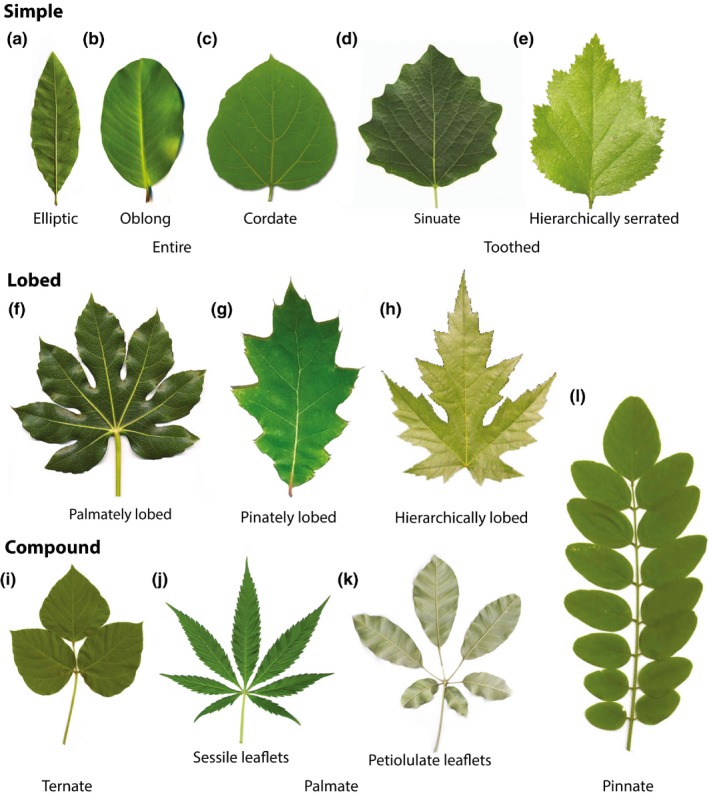
Examples of diverse shapes and features of eudicot leaves. (a) *Quercus imbricaria*. (b) *Garcinia spicata*. (c) *Catalpa bignonioides*. (d) *Populus tremula*. (e) *Crataegus marshallii*. (f) *Fatsia japonica*. (g) *Quercus rubra*. (h) *Acer saccharinum*. (i) *Pueraria montana* var. *lobata*. (j) *Cannabis sativa*. (k) *Handroanthus* sp. (l) *Robinia pseudoacacia*. Photograph sources and credits (see also Supporting Information Notes S1): (a) Jan De Langhe, courtesy of Foundation Arboretum Wespelaar (www.arboretumwespelaar.be); (b) Irène Bouguerra and Nicolas Lagarrigue, Pitchandikulam Forest Virtual Herbarium (www.pitchandikulam-herbarium.org), licensed under CC‐Attribution‐NonCommercial‐ShareAlike 4.0; (c) W. Mark and J. Reimer, courtesy of SelecTree (www.selectree.calpoly.edu); (d) commons.wikimedia.org, licensed under CC‐Attribution‐Share Alike 3.0; (e) John Pickering, courtesy of Discover Life (www.discoverlife.org); (f) commons.wikimedia.org, licensed under CC‐Attribution‐Share Alike 3.0; (h) Renn Tumlison, Henderson State University (www.hsu.edu/Academics/ARNatureTrivia/), used with permission; (i, l) J. K. Marlow, courtesy of Native and Naturalized Plants of the Carolinas and Georgia (www.namethatplant.net); (j) http://commons.wikimedia.org, image in public domain; Karen Blixen (www.flickr.com), licensed under CC‐Attribution‐NonCommercial‐ShareAlike 2.0.

Leaves of eudicots show tremendous morphological diversity (Fig. 1). They can be simple or dissected, that is, partitioned into distinct leaflets, and have margins that are entire (smooth) or have teeth, lobes or sinuses of varying shape and depth (see Supporting Information Fig. S1 for related terminology). In addition, the vascular systems supporting leaf blades may have diverse architectures (Hickey, 1973; Ash *et al*., 1999). Remarkably different leaf morphologies may occur between closely related species, as within‐species variants, or even in the same plant (Kidner & Umbreen, 2010; Nicotra *et al*., 2011). These differences are illustrated by numerous case studies including the leaves of *Pelargonium* (Nicotra *et al*., 2007; Jones *et al*., 2009), grape vine (*Vitis* spp.) (Chitwood *et al*., 2014, 2016), tomato (*Solanum lycopersicum*) (Nuez *et al*., 2004), and the poppy family (Gleissberg, 2004). Diverse leaf shapes also emerge in molecular‐level studies of reference plants including *Arabidopsis thaliana*,* Cardamine hirsuta* and tomato, where small genetic or hormonal changes yield significantly different forms (reviewed by Bar & Ori, 2014; Koenig & Sinha, 2010; Scarpella *et al*., 2010). This lability of shapes, juxtaposed with similar molecular mechanisms underlying leaf development in reference plants, suggests that the striking diversity of eudicot leaves results from variations of a common generative program (Burko & Ori, 2013).

To better understand the essence of this program and examine how it produces diverse forms, we constructed a parametrized computational model of leaf development. The model integrates three perspectives on leaf development: the growth of the leaf blade viewed as a continuous surface, the morphogenetic role of the leaf margin, and the role of the vascular system.

### The continuous surface perspective

The continuous surface perspective has its roots in measurements and a mathematical description of growing tobacco (*Nicotina tabacum*) leaves (Avery, 1933; Richards & Kavanagh, 1943). In present terms, the development of the leaf blade is quantified by a growth tensor field (Hejnowicz & Romberger, 1984), which is formally equivalent to the strain tensor field defined in continuum mechanics. Local growth is integrated into a global description of the developing leaf blade using the mechanical notion of stress–strain relations (Boudaoud, 2010). The continuous‐surface perspective embedded into the computational framework developed by Kennaway *et al*. (2011) was applied to characterize the development of entire *A. thaliana* leaves (Kuchen *et al*., 2012) and winged‐shaped mutant barley (*Hordeum vulgare*) bracts (Richardson *et al*., 2016). The latter model explains the emergence of the lobe‐like wings in terms of polarizers that define different growth directions within a continuous blade.

### Morphogenetic role of the leaf margin

The modelling and understanding of leaf shapes can be facilitated by characterizing them in a more structured way. In particular, the patterning of leaflets, lobes or teeth is largely dependent on the processes that take place at the adaxial–abaxial boundary of a leaf primordium, termed the marginal (leaf) blastozone by Hagemann & Gleissberg (1996). They pointed out that – even in early development, when the leaf primordium is a three‐dimensional bump – the blastozone forms a line that ‘anticipates and circumscribes’, the eventual leaf surface. The blastozone can thus be viewed as a one‐dimensional boundary of a two‐dimensional leaf, similar to the epidermis of a shoot apical meristem being viewed as a two‐dimensional boundary of the three‐dimensional meristem (Floyd & Bowman, 2010; Prusinkiewicz & Runions, 2012; Alvarez *et al*., 2016).

**Figure 2:**
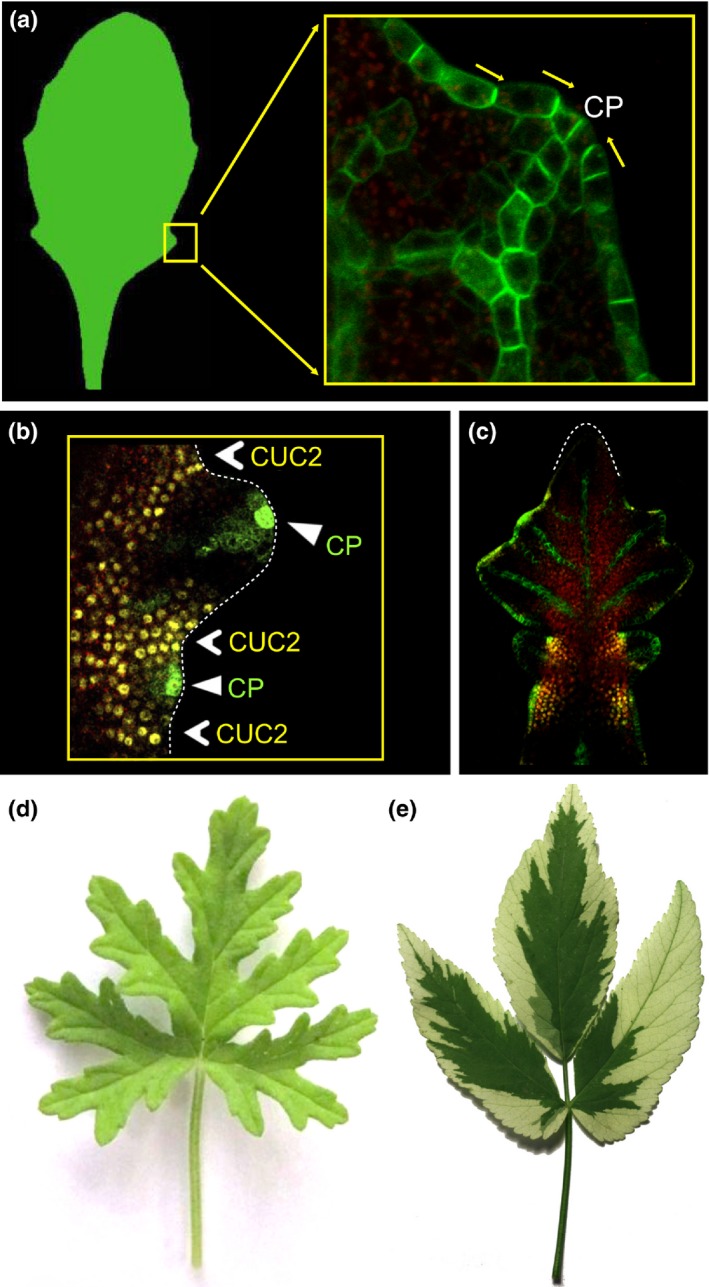
Observations supporting the morphogenetic role of the leaf margin and the alignment of growth axes with veins. (a) Convergence points (CPs) of PIN‐FORMED1 (PIN1) polarization on the margin of an *Arabidopsis thaliana* leaf localize a serration and the tip of a vein. Green, PIN1::PIN1‐GFP. (b) CUP‐SHAPED COTYLEDON2 (CUC2) delimits serrations in *A. thaliana* leaves. Green, DR5::GFP; yellow, CUC2::CUC2‐VENUS. (c) CUC2 marks boundaries between leaflets in a *Cardamine hirsuta* leaf; green, PIN1::PIN1‐GFP; yellow, CUC2::CUC2‐VENUS. (d) Growth of the narrow lobes of a multifid *Pelargonium graveolens* leaf is necessarily aligned with the main veins. (e) Variegation of an *Aegopodium podagraria* leaf indicates the alignment of blade growth directions with the vasculature. Image sources and credits: (a) Hay *et al*. (2006) adapted with permission from *Development*; (b) adapted from Bilsborough *et al*. (2011); (c) image kindly provided by Gemma Bilsborough.

In a growing meristem, feedback between auxin and PIN‐FORMED1 (PIN1) proteins – auxin efflux carriers – leads to the emergence of PIN1 convergence points that position new primordia where space is available for them (Reinhardt *et al*., 2003; Jönsson *et al*., 2006; Smith *et al*., 2006). This feedback provides a molecular implementation of Hofmeister's rule, according to which new primordia emerge at locations that are sufficiently distant from the nearest primordia formed previously (Hofmeister, 1868; see also Kirchoff, 2003). The convergence points also position the endpoints of midveins within the emerging primordia (Bayer *et al*., 2009). In a similar manner, PIN1 convergence points on the margin of *A. thaliana* leaves position leaf serrations (Hay *et al*., 2006) and the endpoints of emerging veins (Scarpella *et al*., 2006) (Fig. 2a,b). This patterning mechanism is largely conserved in other reference plants (Koenig & Sinha, 2010; Scarpella *et al*., 2010; Bar & Ori, 2014; Tameshige *et al*., 2016). The molecular context in which marginal convergence points appear determines whether serrations, lobes or leaflets will develop. For instance, *CUP‐SHAPED COTYLEDON* (*CUC*) genes specify the boundary between serrations in wild‐type *A. thaliana* (Bilsborough *et al*., 2011) (Fig. 2b), but induce lobes by deepening the sinuses when overexpressed (Nikovics *et al*., 2006). In compound *C. hirsuta* leaves CUC2 marks the boundaries between leaflets (Blein *et al*.,^2008^; Hasson *et al*., 2011; Rast‐Somssich *et al*., 2015) (Fig. 2b,c). The postulated interaction between auxin, PIN1 and CUC2 on the growing leaf margin has been included in the computational model of wild‐type and mutant *A. thaliana* leaves (Bilsborough *et al*., 2011). An even simpler model, focused on the feedback between auxin and PIN1 alone, faithfully reproduced lobed ivy (*Hedera* sp.) leaves (Prusinkiewicz & Lane, 2013). In these models, auxin concentration controlled the outward expansion of the leaf margin: faster at the PIN1 convergence points and slower between them. A possible tangential component of growth and the displacement of distal leaf parts by growing proximal parts were ignored. Consequently, the geometry of complex leaf shapes, with a hierarchy of growth axes, could only be captured with limited accuracy (Nakamasu *et al*., 2014).

### Morphogenetic role of the vascular system

Growth directions are inherently accounted for in the third perspective on leaf development, which treats leaves as modified shoots (Arber, 1950). This perspective is related to telome theory, according to which leaves evolved by connecting, or webbing, the free‐standing branching structure of ancestral plants (Zimmermann, 1952; see also Beerling & Fleming, 2007). The local alignment of growth directions with main veins – the present‐day counterpart of ancestral branching structures – is evident in multifid leaves, such as the *Pelargonium graveolens* leaf in Fig. 2(d). Its blade consists of narrow segments, which can only acquire their form by growing faster in the direction of midveins than perpendicular to them. Examination of variegated leaves, in which the dominant directions of growth are indicated by groups of cells with contrasting colours, indicates that they are also aligned with the major veins in leaves with a broad lamina (Dolan & Poethig, 1998) (Fig. 2e). The relative rigidity of veins (Hagemann, 1999; Bar‐Sinai *et al*., 2016) predisposes them mechanically to act as the main axes of growth, with the leaf blade spanning the skeleton of veins. As observed by Dengler & Kang (2001) commenting on an earlier hypothesis by Van Volkenburgh (1999), vascular parenchyma may act as a driver of leaf development, that is, provide the motive force for overall leaf expansion, while mechanical resistance is offered by the epidermis. At the molecular level, the patterning of veins is commonly explained in terms of the canalization theory (Sachs, 1981), which posits that veins differentiate along self‐organizing paths of auxin flow. In this process, a positive feedback between polar auxin transport and the distribution of auxin transporters in cells creates narrow canals of auxin transport in a manner analogous to the carving of rivers by flowing water (Sachs, 2003). The canalization theory is consistent with experimental data and computational models, in which new vascular strands emerge as gradually refined conduits of auxin, connecting convergence points with the existing vasculature (Scarpella *et al*., 2006; Bayer *et al*., 2009; O'Connor *et al*., 2014; Cieslak *et al*., 2015). Models with the aim of capturing the essence of the canalization process at a higher, geometric level of abstraction have also been proposed (Rodkaew *et al*., 2003; Runions *et al*., 2005; Owens *et al*., 2016).

## Description

To better understand the development of leaf form and the basis of leaf diversity, we have constructed a computational model of leaf development that builds upon the three perspectives discussed in the previous section. The model is specified in the L + C extension of the C++ programming language (Karwowski & Prusinkiewicz, 2003). The source code and parameter files are available from the authors’ website (http://algorithmicbotany.org/papers/leaves2017.html). Parameters of all models are also collected in Table S1. Simulations can be executed and visualized using the Virtual Laboratory software environment (http://algorithmicbotany.org/virtual_laboratory). The structure and operation of the model are described in the remainder of this section; further details are given in Notes S2.

### Leaf representation

**Figure 3:**
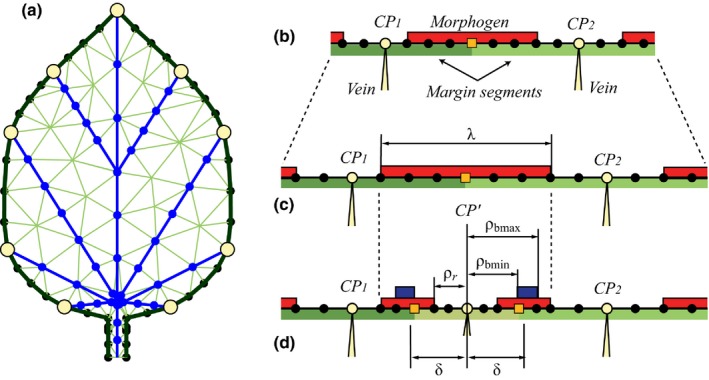
Key elements of the model. (a) Data structures representing a leaf: a polygon representing the leaf margin (black), a tree representing the main veins (blue), and a triangular mesh representing the lamina (green). Yellow circles indicate convergence points, black dots are additional sample points on the margin, and blue dots indicate the vertices at which the mesh coincides with the veins. (b–d) Example of patterning on the margin. (b) Margin fragment with two convergence points, *CP*_1_ and *CP*_2_, and the associated margin segments (green bars). Red bars indicate the presence of a morphogen. (c) Distance relations change as a result of margin growth. An interval with red morphogen reaches threshold length λ. (d) A new convergence point *CP*′ forms in the middle of the red interval and initiates a new vein. Sample points at distance δ or less from *CP*′ become the margin segment associated with *CP*′. The red morphogen is excluded from points closer than ρ_r_ to *CP*′. Another morphogen (blue bars) is introduced at distances greater than ρ_bmin_ and less than ρ_bmax_ from *CP*′.

Consistent with previous models of leaf shape (Bilsborough *et al*., 2011; Prusinkiewicz & Lane, 2013; Nakamasu *et al*., 2014), we disregard the adaxial–abaxial leaf dimension and model the developing leaf as a two‐dimensional structure. We further simplify models by considering development as a planar process, thus ignoring possible three‐dimensional wrinkling and folding of the leaf. We follow Hagemann & Gleissberg (1996) in abstracting from the cellular‐level details of leaf development and focusing on larger components. The leaf is thus represented as three coupled data structures: an open polygon representing the leaf margin (marginal blastozone), a two‐dimensional tree representing the main veins (synonymous with growth axes), and a triangle mesh representing the leaf blade (Fig. 3a). The margin polygon is defined by a sequence of vertices, referred to as the sample points, which begins and ends at the bottom of the petiole. The vascular system is approximated as an open branching structure with straight branches (we do not consider reticulate venation patterns). The root of this system is located at the leaf base, and the terminal points coincide with the convergence points on the margin. The points in the neighbourhood of each convergence point define the margin segment that is associated with this point and with the vein that terminates at it. Each margin point can be characterized further by the presence or absence of one or more morphogens that affect the patterning of subsequent convergence points and/or the growth of the margin (Fig. 3b). Please note that we use the noun ‘morphogen’ as a general term denoting a substance involved in the production of form (Turing, 1952). We do not require that morphogens form gradients.

A sample point is thus specified by its position in space, type (convergence point or not), pointer to the convergence point/vein it is associated with, and one or more flags representing the presence or absence of morphogens. The leaf blade is represented by a triangle mesh, constrained such that all vertices and edges of the leaf margin and the vascular system coincide with vertices and edges of the mesh. During simulation this mesh is dynamically refined to allow for faithful representation of details of the leaf shape as it develops (see Notes S2.1).

### Simulation outline

**Figure 4:**
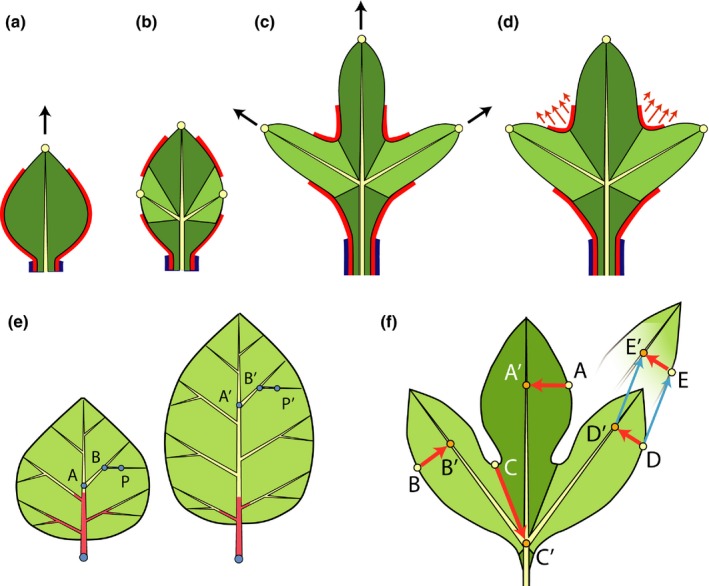
Overview of the model operation. (a–d) Two iterations of simulated development. Coloured lines along the margin indicate morphogens that control development. In this case, the red morphogen enables formation of convergence points and defines sinuses, and the blue morphogen defines the petiole. (a) A leaf at the beginning of the simulation. (b) The midvein grows, driving the expansion of the margin and the blade. New convergence points (yellow circles) emerge on the margin, causing the insertion of new veins and dividing the leaf into regions associated with each vein. (c) Beginning of the next iteration: the veins grow again, causing differential expansion of the leaf in the directions associated with veins. (d) The shape of the margin is further affected by the margin propagation (webbing). (e) Vascular system expansion. Vein elongation is limited to the bottom portion of the leaf (shown in red). This portion does not propagate with the growing leaf and is limited to the vasculature points within the same distance from the leaf base in each simulation step. Vein segments *AB* and *BP* are outside the growth zone and do not elongate (e.g. |*AB*| = |*A*′*B*′| and |*BP*| = |*B*′*P*′|). Although distal parts of the leaf do not grow, they are displaced by the growing bottom part; for instance, point *P* moves to *P*′. (f) Margin growth. Points within a margin segment (e.g. *A*,* B*) are projected perpendicularly onto the corresponding veins (*A*′, *B*′). A boundary point (*C*) is projected onto the branching point between veins (*C*′). Following vein expansion, point *D* is moved by the same vector as its projection: $\vec{DE}=\vec{D^{\prime }E^{\prime }}$.

Leaf form emerges as an outcome of development, simulated as a feedback loop of growth, patterning of the margin and the insertion of new veins. The initial shape of the leaf (leaf primordium with a midvein) and the distribution of morphogens on its margin are specified explicitly at the beginning of the simulation (Fig. 4a). The simulation proceeds iteratively. Each iteration begins with a growth step, driven by expansion of the vascular system. The expansion increases distances measured along the margin and modifies the distribution of morphogens that affect patterning or growth. These changes may trigger the insertion of new convergence points followed by a further modification to the distribution of morphogens (Fig. 4b). The insertion of new convergence points leads to the creation of veins that connect these points to the existing vascular structure and define new growth axes. This step closes the feedback loop: the system is now ready for the next growth step (Fig. 4c,d). The leaf blade interior, spanning the space between the branching vascular system and the leaf margin, expands passively, following the growth of veins and propagation of the leaf margin.

### Growth of the vascular system

Leaf growth is modelled as the superposition of two processes: uniform expansion of the blade, which represents the isotropic component of growth, and growth driven by veins, which introduces an anisotropic component. The growth of veins is modelled, in turn, as the sum of two factors: the addition of new vein segments of a given length at vein tips, and the elongation of existing segments. The latter factor is defined in terms of the relative elementary rate of growth (RERG) (Richards & Kavanagh, 1943; Hejnowicz & Romberger, 1984):

(Eqn 1) $$\text{RERG}\left(P\right)=\frac{\Delta l}{l\Delta t},$$

where *l* is the length of an (infinitesimal) vein segment at point *P*, and Δ*l* is the increase of this segment's length over time Δ*t*. The elongation of a finite‐length vein segment between points *P*_1_ and *P*_2_ is thus the line integral:

(Eqn 2) $$\Delta l_{P_{1}P_{2}}=\int_{P_{1}}^{P_{2}}\text{RERG}\left(P\left(s\right)\right)ds.$$

In general, the RERG may be a highly nonlinear function of position *P* (Kuchen *et al*., 2012). For simplicity, we assume that:

- RERG(P) is a function of the arc‐length distance between point *P* and the leaf base, measured along the vascular structure;
- the above function is piecewise‐linear (we use this assumption to calculate the integral (Eqn 2) analytically); and
- growth preserves vein orientation.

We consider two elongation patterns: uniform (the same rate for all vein segments) and basipetal (growth rates decrease away from the leaf base; Fig. 4e). These patterns are common in eudicots, although other patterns are possible (Jeune & Lacroix, 1993; Das Gupta & Nath, 2015, 2016).

### Margin development

Margin development other than isotropic expansion is driven by growing veins. To this end, the margin is partitioned into segments, each associated with a specific vein. This association is defined dynamically, by assigning a margin interval surrounding a newly created convergence point to the vein that terminates at this point. When the vascular system expands, the margin segments are carried with their associated veins. This process is implemented by projecting points on the margin orthographically on these veins, and translating each margin point by the same vector that describes the displacement of the corresponding vein point (Fig. 4f). As a result, protrusions elongate in the direction of veins. Growth in width is modelled by also propagating margin points in the normal direction (perpendicular to the margin; cf Bilsborough *et al*., 2011). The final component of margin development is a minimization of the stretching and bending of the leaf contour, implemented computationally as geometric fairing (see Notes S2.2). This minimization is intended to reflect mechanical properties of the margin and blade, which may adopt smooth forms when stretched. As stronger minimization leads to shallower sinuses, we refer to it as the strength or rate of webbing.

### Patterning on the margin

**Figure 5:**
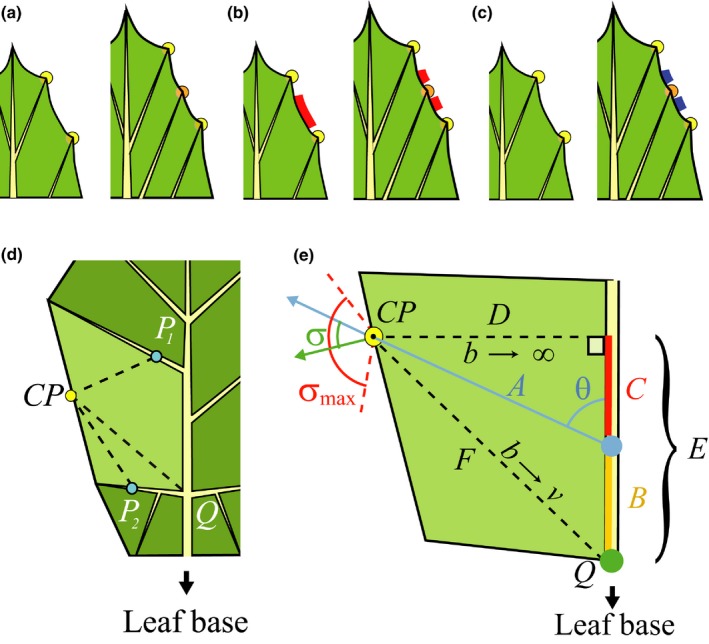
Elements of the model operation: (a–c) patterning on the margin and (d–e) vein insertion. (a) Insertion of a convergence point (orange circle) and the associated vein. (b) Elimination of a morphogen near a new convergence point. (c) Introduction of a morphogen near a new convergence point. Morphogens can determine where convergence points can be inserted. For example, in case (b) the CP can be inserted where the morphogen is present, and in case (c) it can be inserted where the morphogen is absent. (d) Potential attachment points of the new vein. A region of leaf lamina is bordered by the leaf margin and vein segments. A new vein, originating at convergence point *CP*, may attach to an existing vein (*P*_1_, *P*_2_) or a branching point between the existing veins (*Q*). (e) Calculation of vein direction. With *b* → ∞, the new vein *D* meets an existing vein at a right angle. With *b*→*v*, the new vein *F* attaches to the branching point *Q* between existing veins. For intermediate values *b*, the new vein *A* may attach to a pre‐existing vein at some angle θ. Symbols *A* to *F* represent the lengths of the respective segments in the derivation for a formula for θ (Supporting Information Notes S2.3). The angle σ between the new vein and the normal to the margin is clamped to ±σ_max_.

Consistent with the extension of Hofmeister's rule to the leaf margin, new convergence points are created at margin positions that exceed a threshold arc‐length distance (measured along the margin) from pre‐existing convergence points or the leaf base (Fig. 5a). The introduction of new convergence points may be limited to regions in which specific morphogens are – or are not – expressed, to leaf parts within a certain distance from the leaf base (measured along the veins), and/or to a temporal competence window defined by leaf age. The initial morphogen distribution is determined by the modeller at the beginning of the simulation. The introduction of a convergence point may modify this distribution by eliminating or introducing new morphogens in its proximity (Figs 3b–d, 5b,c). In addition to affecting the creation of new convergence points, morphogens typically regulate the rate of webbing and may locally modify the measure of distances between convergence points. A segment of the margin surrounding a newly formed convergence point becomes a part of the incipient protrusion.

### Vein insertion

Consistent with molecular data (Scarpella *et al*., 2006; Bayer *et al*., 2009; O'Connor *et al*., 2014), a new convergence point on the leaf margin induces a vein that connects this point to the existing vasculature. The attachment point *P* at which this vein will meet the vasculature is computed using two heuristics (Fig. 5d). The first heuristic is motivated by the observation that vascular strands tend to provide short and straight connections between sources and sinks (Bayer *et al*., 2009), and the hypothesis that these connections may minimize resistance to the transport of water and photosynthates in the leaf (Sack & Scoffoni, 2013). According to this heuristic, the attachment point *P* is found by minimizing the expression τ = *b*|*CP*−*P*| + *v*|*P*−*B*|, where |*CP*−*P*| is the length of the inserted vein (the distance between the new convergence point *CP* and the attachment point *P*), |*P*−*B*| is the length of the path from the attachment point to the leaf base *B* (the arc‐length distance from *P* to *B*, measured along the veins), and parameters *b *≥ *v* > 0 are the resistances to transport per unit distance in the leaf blade and in the veins, respectively. Variable τ thus represents the total resistance to transport from the convergence point to the leaf base. It can be shown that any vein minimizing τ meets an existing vein at a constant branching angle θ = arccos(*v*/*b*) (Notes S3) or is attached to an existing branching point. Assuming that resistance to the transport of water and sugars is anticipated by resistance to the transport of auxin that patterns the vascular system, the resistance‐based model is qualitatively supported by the observed reduction of the angle at which secondary veins meet the midvein in plants treated with an auxin transport inhibitor (Mattsson *et al*., 1999). The second heuristic constrains the angle between the new vein and the normal to the margin to an interval [−σ_max_, σ_max_]. It prevents veins from approaching the margin at unnaturally small grazing angles.

## Results

The parameter values for the leaf forms and developmental sequences discussed in this section are listed in Table S1. These values were found in two types of *in silico* experiment. In the first type (theoretical morphospace exploration; McGhee, 1999), we systematically modified one or two parameters while keeping the remaining parameters constant to visualize the space of resulting forms. In the second type (synergistic human‐computer problem solving; Licklider, 1960), we modified and refined parameter values interactively to approximate select real leaves. When needed, this process also involved adjustments to the model code, gradually yielding the current implementation.

### Plausible patterns of leaf development emerge from a self‐organizing process

**Figure 6:**
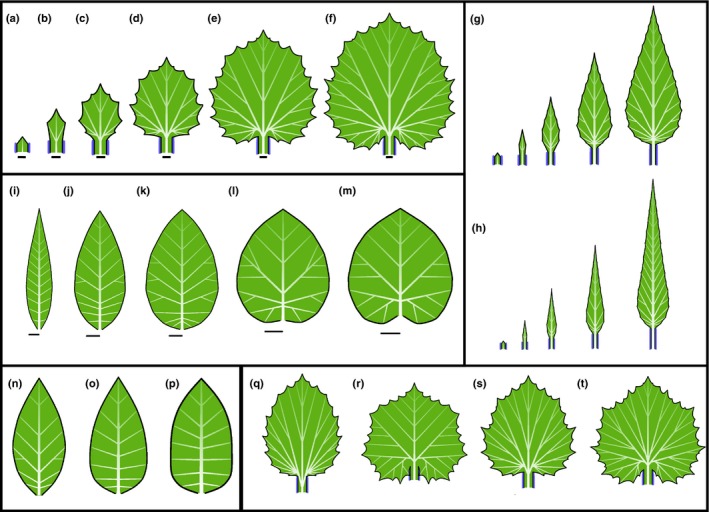
Self‐organizing development of simple leaves and the role of selected parameters. (a–f) Simulation of the development of a generic simple leaf (bars indicate constant reference length). The leaf is initiated as a small primordium, with a single convergence point at its apex (a). The midvein connects this convergence point to the leaf base and determines the initial direction of growth. As the leaf grows, the increasing distance from the leaf base to the tip, measured along the margin, leads to the emergence of new convergence points and the lateral veins associated with them (b). Further convergence points and veins subsequently emerge, gradually expanding the leaf in the lateral (c, d) and basal (e, f) directions. The blue morphogen delineates the petiole. Bars indicate the relative size of the leaf at different developmental stages. (g, h) The impact of different growth rates on leaf shape. (g) Moderately and (h) strongly reduced marginal growth of lateral veins, compared with the sequence (a–f), produces elongated leaves. (i–m) Impact of vein growth at the tip on leaf shape. The growth at the vein tip increases from leaf (i) to (m), producing increasingly broad leaves (bars indicate constant reference length). The emergent vascular pattern changes from pinnate (with a single main vein) to hierarchically branching. Total growth duration decreases from (i) to (m). (n–p) The impact of growth distribution. With the growth increasingly limited to the basal portion of the leaf, the leaf form shifts from elliptic (n) to ovate (o) to oblong (p). (q–t) The impact of parameters controlling the insertion angle of new veins on leaf form. The branching angle θ is smaller in (q) and larger in (r) than in the reference leaf (e). Decreasing the range σ_max_ of admissible angles between the vein and the normal to the margin results in more varied vein directions (s, t).

A basic pattern of development produced by the model is illustrated in Fig. 6(a–f) (Movie S1). A heart‐shaped leaf with branching venation is produced in a self‐organizing process, in which convergence points determine veins, veins determine growth directions, and growth of the margin leads to the formation of new convergence points. The only leaf part excluded from this loop is the petiole, where a locally expressed morphogen suppresses lateral growth and inhibits the formation of convergence points. The overall form is not defined globally, but results from the integration of local subprocesses.

### Growth distribution controls both the shape and venation pattern of simple leaves

The impact of additive marginal growth at vein tips is illustrated in Fig. 6(g,h,i–m). If the marginal growth is small, the vascular system expands uniformly. Lateral veins, inserted after the midvein, are relatively short, and this proportion is maintained throughout the subsequent growth of the leaf. The result is an elongated leaf with a strictly or approximately pinnate venation (Fig. 6g,h,i,j). A uniform increase in the marginal component of growth gradually reduces the relative differences in vein lengths, producing leaves with broader blades (Fig. 6k,l). With even stronger marginal growth, higher order veins emerge near the leaf base, yielding cordate leaf forms (Fig. 6f,m).

Limiting growth to basal portions of the leaf prevents elongation of veins in more distal positions, which results in a transition of leaf shape from elliptic to ovate to oblong (Fig. 6n–p). The aspect ratio (width: length) of a leaf also depends on the branching angle θ between the veins: as this angle increases, the leaf becomes wider (Fig. 6q,r). Decreasing the range σ_max_ of angles that a vein can form with respect to the margin prevents veins from reaching the margin tangentially (compare the veins near the base of the leaf in Fig. 6q with those in Fig. 6s,t). If clamping to ±σ_max_ overrides other criteria of vein insertion, small values of σ_max_ may induce a variation in the branching angles θ between veins. This variation is reflected in variable vein orientations and a less regular leaf margin (Fig. 6s,t).

### Differences in webbing control the margin of simple leaves

**Figure 7:**
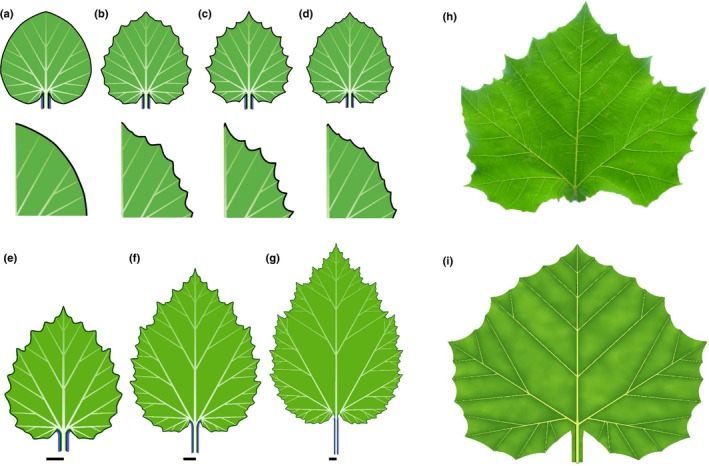
Control of the leaf margin. (a–d) Webbing and the shape of protrusions. The differences in the protrusions are highlighted by zooming in on the margin (second row). Strong webbing produces an entire (smooth) leaf margin (a). Weaker webbing produces sinuate margins when the resistance to bending is relatively strong (b), and dentate margins, with pointed teeth, when the resistance to bending is weaker (c). Asymmetry in the influence of veins on their proximal and distal sides produces serrations pointing towards the leaf apex (d). (e–g) Emergence of compound teeth. As the expansion of the leaf becomes more uniform from (e) to (g), the form of teeth progresses from simple to compound (bars represent the same length). (h, i) Example of model application: a photograph (h) and model (i) of a *Platanus occidentalis* (sycamore) leaf with compound teeth. Following the primary morphogenesis responsible for the patterning of protrusions and veins, the simulated leaf was assumed to expand anisotropically (faster in width than in length) to achieve the correct aspect ratio (width : length > 1). Photograph (h) by Brian Bale (www.treeplantflowerid.com), used with permission.

Webbing plays a critical role in defining the leaf margin. Strong webbing, characterized by a significant resistance to stretching and bending, results in a smooth margin (Figs 6i–p, 7a). With reduced webbing, the parts of the margin further from the extending tips lag behind those near the tips, resulting in the formation of teeth. The shape of these teeth depends on the resistance of the leaf margin to bending (Fig. 7b,c). Different resistance for stretching at the proximal and distal segments of the polygonal approximation of the margin, meeting at a convergence point, results in asymmetric serrations (Fig. 7d).

### Uniform expansion promotes the emergence of compound teeth

In the examples discussed so far, different parts of the leaf blade expand anisotropically following the growth of veins in their proximity. A margin segment spanning the endpoints of two parallel growing veins may thus not expand at all. By contrast, an isotropic expansion of the entire leaf increases all distances along its margin uniformly. This may lead to the recursive insertion of intercalary convergence points between those formed previously, inducing a hierarchy of teeth (Fig. 7e–g; Movie S2). Similar hierarchies occur in many leaves in nature; for instance, compare the model in Fig. 7(g) with the photograph of a *Crataegus marshallii* (parsley hawthorn) leaf in Fig. 1(e). Another example is given in Fig. 7(h,i), which compares a photograph and model of a *Platanus occidentalis* (American sycamore) leaf. Note the similarities in both the shape of the leaf and the structure of its vascular system.

### Inhibition of convergence point formation in sinuses yields leaves with lobes

**Figure 8:**
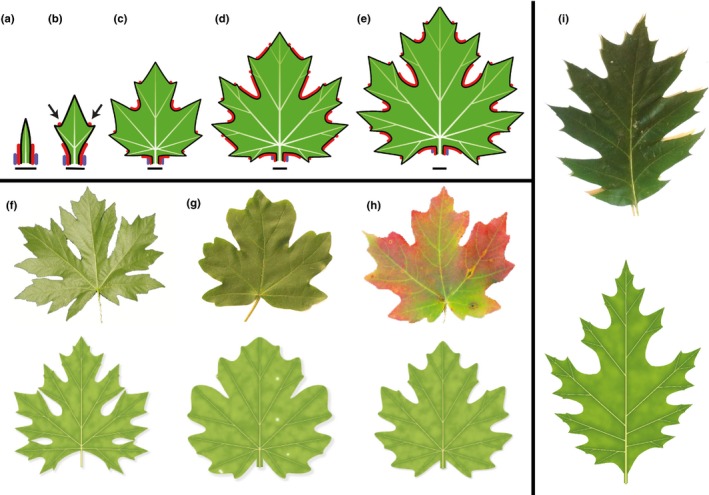
Modelling lobed leaves. (a–e) Simulation of the development of a generic palmately lobed leaf (bars indicate constant reference length). The red morphogen inhibits the formation of intercalary convergence points and deepens the sinuses. (a) At the beginning of the simulation, the red morphogen is present near the base of the incipient leaf. (b) As the leaf grows, new convergence points emerge in the morphogen‐free intervals, and new intervals of the red morphogen form between the old and new convergence points (arrows). (c–e) Iteration of this process elaborates leaf shape while preventing the formation of new convergence points in the sinuses. As in the previous examples, the blue morphogen defines the petiole. (f–h) Modelling leaf diversity: photographs and models of (f) *Acer macrophyllum* (big leaf maple), (g) *Acer campestre* (field maple) and (h) *Acer grandidentatum* (bigtooth maple). All models result from small changes in the parameter values of the generic palmate leaf model illustrated in (e). (i) A photograph and model of a pinnately compound *Quercus rubra* (northern red oak) leaf. The transition from palmately to pinnately compound form results from a larger window of morphogenetic competence for the red morphogen, enabling the insertion of primary lobes further from the leaf base, compared with the palmately lobed leaves. Photograph sources and credits: (f) Dan Anderson (www.tree-species.blogspot.com), used with permission; (g) Middle European Woods data set (Novotný & Suk, 2013) (http://zoi.utia.cas.cz/node/662), licensed under CC‐Attribution‐ShareAlike 3.0; (h) Adapted from a photograph by Dean Hueber (http://www.pbase.com/deanhueber/image/90024562; downloaded 20 July 2016).

Decreasing the strength of webbing deepens the indentations between teeth. If, in addition, a morphogen inhibits the formation of new convergence points in these indentations, a lobed leaf results (Fig. 8a–e; Movie S3). The formation of new convergence points, and thus new veins and higher order lobes, is then limited to the distal (subapical) part of each lobe. With different parameter values, this model captures the shape of many common leaves. For instance, Fig. 8(f–h) shows the palmately lobed leaves of three maple species.

In Fig. 8(a–h), first‐order lobes are initiated close to the leaf base. If the window of morphogenetic competence is moved upward from the leaf base, a more elongated leaf blade supported by a pinnate vascular system results (Fig. 8i). A similar dependence of leaf type (palmate vs pinnate) on the position of the window of morphogenetic competence was observed in the model study by Jeune & Lacroix (1993).

**Figure 9:**
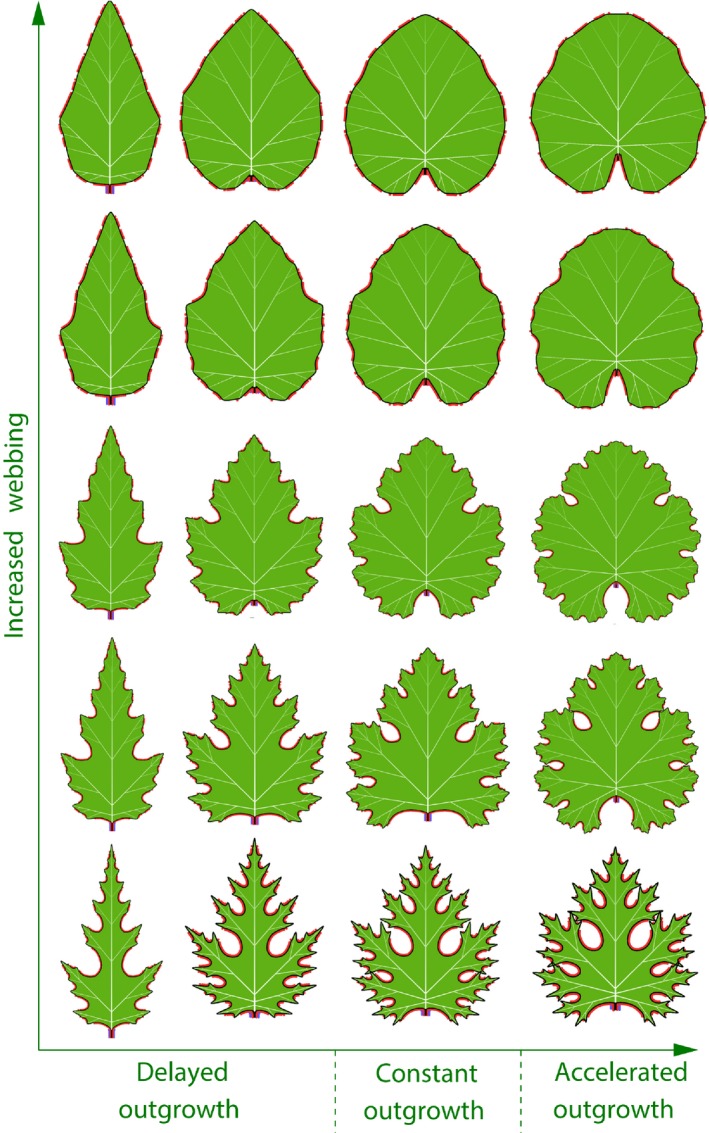
Morphospace of leaves controlled by the timing of lateral outgrowth and the rate of webbing. The action of the red and blue morphogen is as in Fig. 8. With accelerated outgrowth, leaves become more rounded, and the vascular pattern gradually changes from pinnate to palmate. A decrease in webbing increases the depth of sinuses. The resulting increase in margin length leads to the emergence of additional convergence points, and the simulated shapes shift from entire to recursively lobed.

Elongated pinnately lobed leaves also emerge when the growth of lateral veins is delayed with respect to the insertion of convergence points that induce these veins. Conversely, broader palmately lobed leaves emerge if the growth of lateral veins is accelerated (Movie S5). The morphospace in Fig. 9 illustrates the combined effect of this delay/acceleration and the rate of webbing. The latter factor controls a progression of leaf shapes from simple to recursively lobed. Select forms in this morphospace resemble leaves of different *Pelargonium* and *Chrysanthemum* species, although the full spectrum of their diversity is larger (Jones *et al*., 2009; Kim *et al*., 2014).

### Spatio‐temporally limited competence to create convergence points yields leaves with simple lobes

**Figure 10:**
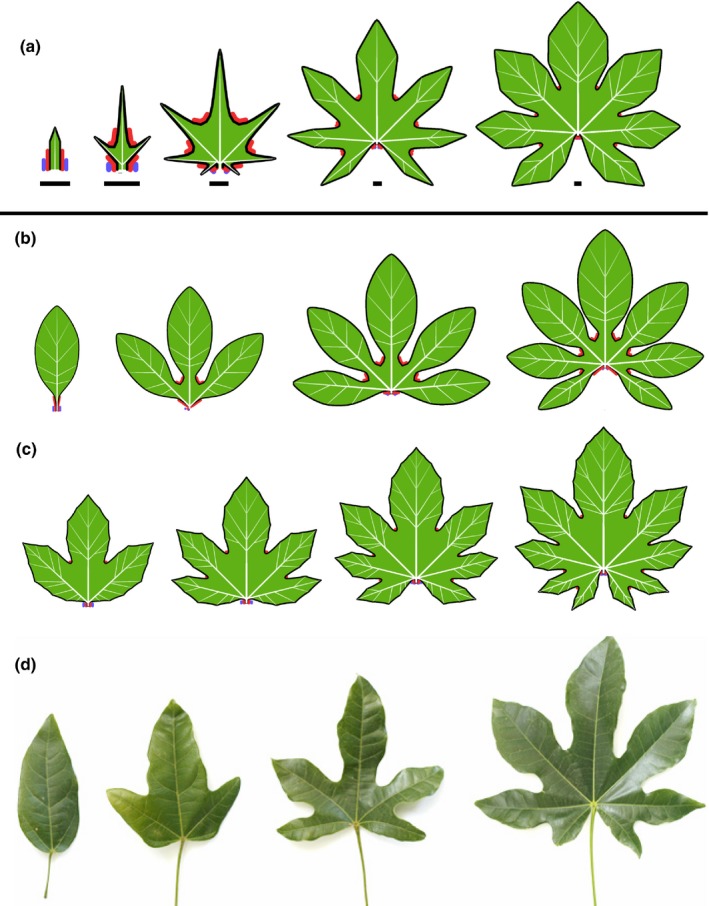
Modelling palmately lobed leaves with simple lobes. (a) Development of a generic leaf (bars indicate constant reference length). The red morphogen acts in early stages of development by deepening the sinuses and decreasing the measure of distances, which fosters the early initiation of several lobes. The blue morphogen delineates the petiole. Secondary veins form later, when the leaf is relatively large, without adjacent growth repression. The role of these veins is limited to broadening the lobes. (b–d) Variation in the number of lobes resulting from different temporal competence for lobe formation. Two sequences, with (b) stronger and (c) weaker action of the morphogen controlling growth in the sinuses (red), are shown. Similar variations are observed in *Brachychiton acerifolius* (flame tree) leaves (d), in which one to seven lobes occur apparently at random within the same tree.

**Figure 11:**
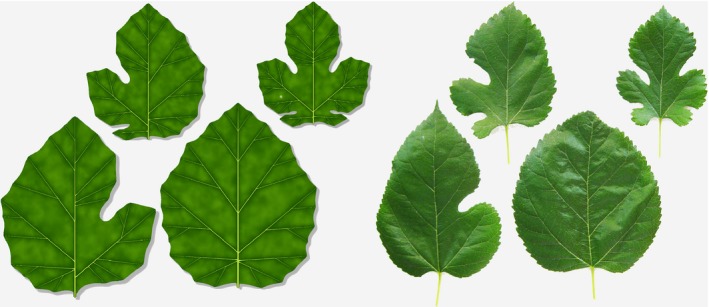
A model and photograph of different mulberry (*Morus alba*) leaves originating from the same plant. The model is similar to that illustrated in Fig. 10. Asymmetries are attributable to the initial unequal distribution of morphogens on the left and right sides of the leaves. Adapted from a photograph by Evelyn Fitzgerald (https://www.flickr.com/photos/evelynfitzgerald/3917066690/sizes/l, downloaded 2 July 2016).

The progression from leaves with compound teeth (Fig. 7e–i) to leaves with compound lobes (Figs 8, 9) was modelled by introducing a morphogen that suppressed the emergence of convergence points and growth in sinuses. An additional developmental control is needed to suppress the formation of higher order lobes. It can be effected by a spatio‐temporal window of morphogenetic competence that limits the formation of sinuses to early stages of leaf development and to locations near the leaf base (Fig. 10a; Movie S5). Small changes in the duration of competence yield leaves with different numbers of lobes (Fig. 10b,c). The simple, quantitative nature of these changes may explain the lability of lobe numbers common in some plant species, for example *Brachychiton acerifolius* (flame tree; Fig. 10d). Moreover, small differences in the initial development of the left and right sides of the leaf, for example as a result of differences in the distribution of morphogens, may be amplified by further development producing asymmetric leaves with different numbers of lobes on the left and right sides (Fig. 11).

### Strong inhibition of webbing in sinuses yields dissected leaves

**Figure 12:**
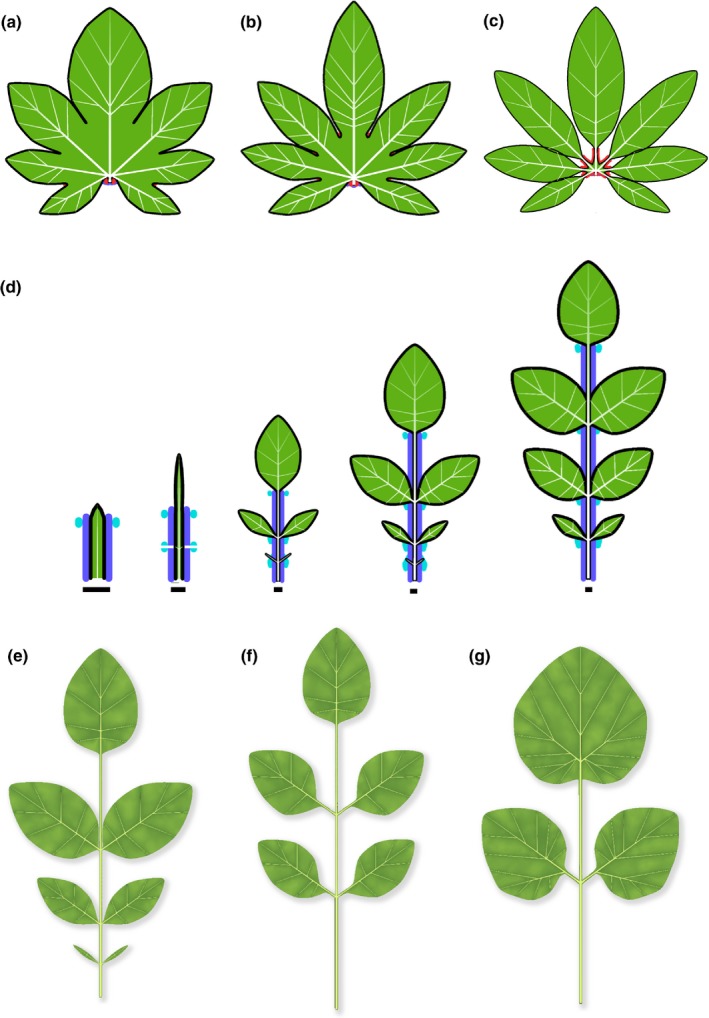
Modelling compound leaves. (a–c) Transition from a palmately lobed to a palmately compound leaf. The increasingly strong action of the red morphogen decreases webbing of sinuses, resulting in a progression of leaf forms from (a) moderately to (b) strongly indented palmately lobed leaves to (c) a palmately compound leaf. (d) Development of a pinnately compound leaf (bars indicate constant reference length). The purple morphogen inhibits webbing, which results in the formation of a linear petiole and rachis. The cyan morphogen delimits the leaflets at their base and divides the leaf margin into intervals. Distances are measured independently within each interval, thus separating the development of individual leaflets from the development of the rachis. (e–g) Variations of the compound leaf form from (d). Leaflets are sessile (attached directly to the rachis) in leaf (e), and supported by petiolules (small petioles) in leaves (f) and (g). The leaflets in (f) and (g) are slightly asymmetric as a consequence of a difference in the initial position of the morphogens on the lower (proximal) and upper (distal) sides of the leaflet primordia relative to the supporting vein.

Increasing the inhibition of webbing by a morphogen acting in sinuses shifts leaf form from moderately to strongly lobed to palmately compound (Fig. 12a–c). Development of pinnately compound leaves is more complex. Not only must the webbing be suppressed along the midvein to produce the petiole and rachis, but the elongation of the rachis must also be suppressed at the points of leaflet attachment to prevent excessive widening of the leaflet bases. These requirements can be satisfied by the coordinated action of two morphogens (Fig. 12d; Movie S6). The first morphogen suppresses lateral growth in sinuses, as in Fig. 12(c). It does not prevent, however, the formation of new convergence points. The growth of the petiole and rachis can thus induce new convergence points and leaflets. The second morphogen is generated near each convergence point and defines the leaflet boundary at its point of attachment to the rachis. It inhibits longitudinal growth of the rachis, as required to properly form leaflet bases, and divides the margin into intervals within which distances are measured independently. This division stabilizes the development of leaflets by isolating them from the processes that initiate new leaflets along the midvein. Through varying model parameters, both sessile and nonsessile leaf forms arise (Fig. 12e,f). In addition, the model can capture subtle asymmetries in leaflet shape along the proximo‐distal leaf axis, as a result of slightly different initial conditions on the two sides of the petiolules (Fig. 12g).

## Discussion

The central question addressed in our paper is the developmental origin of leaf diversity. Following the inferences of Hagemann & Gleissberg (1996), molecular data, and previous computational models outlined in the Introduction, we have attributed patterning of protrusions and indentations to morphogenetic processes taking place on the leaf margin. Furthermore, based on observations of multifid and variegated leaves, and taking into account the mechanical rigidity of veins, we hypothesized that main veins play an important morphogenetic role by defining local growth directions, that is locally polarizing growth. Consistent with the hypotheses of Van Volkenburgh (1999) and Dengler & Kang (2001), we also assumed that the intervening leaf blade tissue locally follows these directions. With different parameters, our model captures the essential aspects of the development and shape of a wide range of eudicot leaves, which supports its plausibility and leads to the following conclusions.

### Leaf development is a self‐organizing process

**Figure 13:**
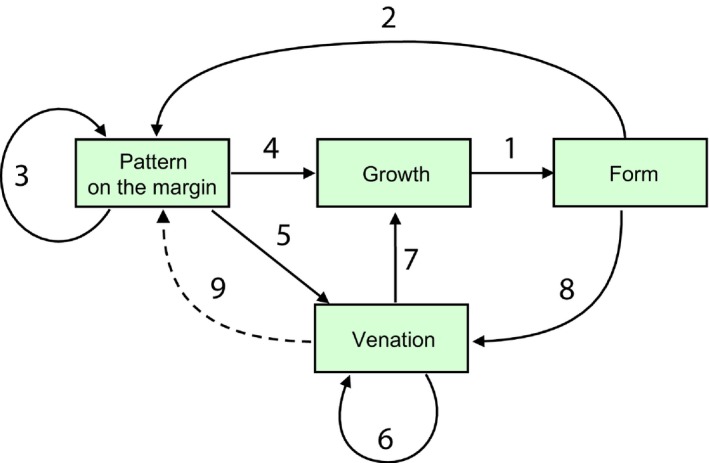
Relation between processes underlying leaf development. (1) Growth yields form. (2) Changes in the margin geometry induce new convergence points and modify the distribution of morphogens. (3) Existing convergence points and morphogens provide the context in which changes take place. (4) Convergence points and morphogens control the rates of leaf growth. (5) Convergence points induce veins. (6) The course of new veins is affected by the existing vasculature. (7) Veins specify local growth directions. (8) The geometry of the vascular system changes as the leaf grows. (9) Hypothetically, veins may also affect the induction of convergence points, although this influence is not included in the present model.

Molecular‐level processes apparently act by establishing the ‘rules of the game’ that integrate growth, dynamic patterning on the leaf margin, and the formation of the vascular pattern into a self‐organizing system characterized by several feedback loops (Fig. 13). In particular, auxin‐driven interactions on the leaf margin establish a metric (distance measure) for patterning protrusions and indentations, and the vascular system complements the morphogenetic role of the margin by specifying local growth directions.

### A common mechanism can produce widely diverse leaf forms

It is known that small modifications to a self‐organizing process can fundamentally change its outcome (Wolfram, 1984). This does not preclude different molecular implementations of the same developmental program, or, conversely, the recruitment of the same molecular process for different morphogenetic purposes. The self‐organizing character of leaf development is probably essential to the diversity of leaf forms. For example, the frequently observed transitions between simple, lobed and recursively lobed leaves (e.g. Hareven *et al*., 1996; Hay & Tsiantis, 2006; Jones *et al*., 2009; Efroni *et al*., 2010; Bar & Ori, 2014) (Fig. 9) may be attributed to the feedback loop in which weaker webbing produces deeper sinuses, and the resulting increase in the length of the leaf margin creates space for new convergence points, veins and lobes. Such changes can be plausibly attributed to small modifications of the plant genotype, differences in developmental context (heteroblasty), or environmental factors (Nicotra *et al*., 2011).

### Hofmeister's rule extends to leaf development

The insertion of new convergence points when the distances to previously formed points exceed a threshold plays a prominent role in leaf development. In the context of phyllotactic patterning, this distance‐based criterion is known as Hofmeister's rule. Hofmeister noticed that ‘the appearance of new lateral organs in the largest of the spaces between the nearest older organs of the same type on the same axis is a phenomenon of almost complete universality’ (quoted after Kirchoff, 2003). We observe that the universality of this rule exceeds even its author's expectations: it applies to the emergence of new outgrowth not only ‘on the same axis’, but along the leaf margin as well.

The extension of Hofmeister's rule from phyllotaxis to leaf formation has its source in the similarities between the molecular processes governing the two phenomena. They include the creation of convergence points through the interaction between PIN1 and auxin, the role of additional morphogens, such as CUC proteins, as factors shaping boundaries, and the formation of vascular strands by auxin‐driven canalization beginning at convergence points (Floyd & Bowman, 2010; Alvarez *et al*., 2016). These similarities may reflect the common nature of the developmental problem solved by plants in each case: how to place a number of elements (flowers, floral organs, leaves, leaflets and lobes) within the constraints of available space. The strikingly different appearance of spiral phyllotactic patterns and leaves results ‘not from fundamentally different morphogenetic processes, but from different geometries on which they operate: an approximately paraboloid SAM (shoot apical meristem) dynamically maintaining its form vs a flattening leaf that changes its shape and size’ (Prusinkiewicz & Runions, 2012).

### The telome theory and the notion of blastozone are related to each other

Zimmerman's telome theory postulates that leaves evolved by webbing the branching structures of early land plants. It does not specify, however, whether the control of development has remained with the branching structure – corresponding to the leaf vasculature – or has been transferred to another part of the leaf. Hagemann and Gleisberg's blastozone theory implies that the control of development has been transferred to the leaf margin. Over the last decade, it found strong experimental support in molecular studies of reference plants. From an evolutionary perspective, the transfer of control from the branching structure to the leaf boundary has the advantage of creating a vascular scaffolding in concert with the available space on the leaf margin. This phenomenon can be observed, for example, in the gradual proliferation of veins near the base of cordate leaves (e.g. Figs 6a–f, 7h,i) and the emergence of small veins supporting higher order protrusions in recursively lobed leaves (compare leaves in the right column of Fig. 9).

### Geometric models provide a framework for interpreting molecular mechanisms of leaf development

Geometric terms are an abstraction that highlights the morphogenetic role of specific molecular‐level processes (Prusinkiewicz & Runions, 2012; Runions *et al*., 2014). For instance, the interaction between auxin and PIN proteins in the epidermis of the shoot apical meristem or on the leaf margin can be characterized as a mechanism for distance measurement. This characterization furnishes an explanation for the initiation of convergence points in regular patterns (Jönsson *et al*., 2006; Smith *et al*., 2006; Bilsborough *et al*., 2011; O'Connor *et al*., 2014), although additional biomechanical (Hamant *et al*., 2008) or biochemical factors (e.g. CUC proteins; Bilsborough *et al*., 2011) are also relevant.

The current understanding of CUC proteins suggests that they are involved in shaping the boundaries of protrusions, such as serrations in *A. thaliana* leaves (Nikovics *et al*., 2006; Blein *et al*., 2008; Bilsborough *et al*., 2011), or leaflets in the compound leaves of tomato (Berger *et al*., 2009) and *C. hirsuta* (Rast‐Somssich *et al*., 2015). However, CUC also plays an important role in enabling the formation of convergence points (Bilsborough *et al*., 2011). This role was not conserved in all the models devised in our work, suggesting that the coordination between the initiation of protrusions and the sculpting of the indentations between them may be more diversified than studies of current reference plants indicate.

Other genes and proteins can also be interpreted in the context of our models. The recently discovered growth inhibitor REDUCED COMPLEXITY (RCO) contributes to leaf dissection (Sicard *et al*., 2014; Vlad *et al*., 2014) and appears to play a critical role in defining linear elements – the rachis and petiolules – of compound leaves (Vlad *et al*., 2014). A similar role may be played by several auxin response factors (ARFs) in tomato (Ben‐Gera *et al*., 2016). The development of compound leaves also involves class I *KNOTTED‐like homeobox* (*KNOX*) genes (Bharathan *et al*., 2002; Bar & Ori, 2014), which delay the progression of leaf maturation, and appear to extend the spatio‐temporal window of marginal patterning. By contrast, *TB1 CYCLOIDEA PCF* (*TCP*) genes (Bar & Ori, 2014) accelerate differentiation and reduce the duration of marginal pattering, at least in part by antagonizing CUC activity (Koyama *et al*., 2010; Rubio‐Somoza *et al*., 2014). Accordingly, decreasing *TCP* expression delays maturation and increases the number of protrusions initiated at the leaf margin (Barkoulas *et al*., 2007; Bar & Ori, 2014). With research in progress on the molecular underpinnings of leaf shape, computational modelling is likely to continue to play a significant role in verifying whether the spatio‐temporal patterns of gene expression and morphogen distribution are consistent with the morphogenetic roles attributed to them, synthesizing our understanding of leaf development, and providing a framework for considering this development from an evolutionary perspective.

### Open problems

The mechanism by which the veins extending from a convergence point find their target location is unclear (Bayer *et al*., 2009) and, consequently, so is the developmental mechanism that determines the branching angles between veins of different orders. The heuristics used in this paper provide a working hypothesis, but also highlight the need for research exposing the molecular mechanisms through which veins find their attachment point and create particular branching angles. Moreover, the assumption of an open venation system is conspicuously violated in brochidodromous patterns, in which secondary veins form pronounced loops. Although some models can create closed loops (Couder *et al*., 2002; Runions *et al*., 2005), the mechanisms that control the development of closed venation patterns remain largely unknown (Scarpella *et al*., 2010). Even in open venation systems, the assumption of straight veins is a simplification, as different processes – for example, nonuniform expansion of the lamina with the embedded veins – may yield curved veins. In addition, some leaves include veins that penetrate indentations rather than protrusions. A detailed investigation of the paths of veins and the mechanisms that produce them remains an intriguing open problem, bridging molecular biology and differential geometry.

Our model postulates that growth directions are locally aligned with the veins. With veins running in different directions, as dictated by their branching pattern, the growth directions and magnitudes may form a highly nonuniform field. A detailed multiscale analysis of growing leaves is needed to reveal whether this mathematical possibility holds for real leaves. The growth tensor would then be scale dependent, in the same way as distance measures are scale dependent in fractal objects. The fractal nature of the growth field may be obscured when considering leaves at larger scales. This would explain why the average values of the growth tensor inherent in continuous‐surface models suffice when characterizing and modelling simple or broadly lobed leaves (Kuchen *et al*., 2012; Richardson *et al*., 2016).

Recent experimental results obtained by Das Gupta & Nath (2016) indicate that different leaf growth patterns, at a coarse level manifested by the distinction between basipetal, uniform and acropetal growth, occur across eudicot species, contributing to the diversity of their leaves. We have acknowledged this source of diversity in our paper (Fig. 6n–p), but its interplay with other morphogenetic factors and impact on the final leaf forms is presently unclear and awaits further study.

Our model ignores departures of leaves from planarity. The molecular basis of leaf curvature – for example a delayed arrest of growth near the leaf margin – is increasingly well understood (Nath *et al*., 2003; Sharon *et al*., 2007; Efroni *et al*., 2008; Prusinkiewicz & de Reuille, 2010), but modelling the morphogenesis of leaves that are curved as well as lobed or serrated remains an open problem. Extending the model described in this paper to curved leaves would probably entail incorporating a biomechanical component, as their forms can most readily be expressed as states of minimum energy of surfaces resisting stretching and bending. Challenges are posed by the nonhomogeneous structure of leaves, with veins embedded within a thin leaf blade (Hong *et al*., 2005), and the need to replace straight vein segments with their more complicated counterparts defined on curved surfaces: the geodesic curves.

Many leaves are folded as they develop. This folding may play an important morphogenetic role, especially in the case of leaves that develop within the confines of a bud (Couturier *et al*., 2009, 2011, 2012). Reconciling models described in this paper with those attributing a significant morphogenetic role to folding remains an open problem, although a link between them can be foreseen (for instance, convergence points may define how the leaf is folded).

While the leaves discussed so far can be approximated as flat or curved surfaces (formally, two manifolds), some succulent leaves are fully three‐dimensional, volumetric structures (three manifolds) and have a correspondingly three‐dimensional vasculature (Korn, 2011; Ogburn & Edwards, 2013). It would be most interesting to determine whether our model can be extended to this case as well, possibly shedding light on the evolutionary path between surface‐like and volumetric leaves.

Finally, our models are supported by visual comparisons of generated forms with photographs of mature leaves. This brings into focus the lack of adequate data concerning the development of diverse leaves from the earliest stage of leaf primordia to maturity. Acquiring such data using current methods is a tedious process (cf. Kuchen *et al*., 2012); however, recent results indicate that the diversity of growth patterns is not well represented by the limited spectrum of current reference plants (Das Gupta & Nath, 2015, 2016). In addition, visual comparisons should be complemented by measurements and comparison criteria rooted in leaf morphometrics (for example, see Biot *et al*., 2016 and references therein). An examination of models in light of quantitative data will provide an opportunity to refine the models and validate them objectively; conversely, we expect that the models will provide a useful theoretical framework for interpreting new experimental results.

## Author contributions

P.P. and A.R. designed the research; A.R. performed the research; P.P., A.R. and M.T. analyzed and interpreted the results; P.P. and A.R. wrote the paper with input from M.T.

## Supporting information

Please note: Wiley Blackwell are not responsible for the content or functionality of any Supporting Information supplied by the authors. Any queries (other than missing material) should be directed to the *New Phytologist* Central Office.

**Fig. S1** Selected terms pertinent to leaf morphology.
**Notes S1** Image sources and credits.
**Notes S2** Additional details regarding the implementation of the generative model of leaf form development.
**Notes S3** A proof of the relationship between resistance and branching angle given in the main text.

**Table S1** Parameter values used in simulations

**Movie S1** Simulation of the development of a generic simple leaf (corresponds to Fig. 6a–f).

**Movie S2** Simulation of the development of a leaf with compound teeth (corresponds to Fig. 7g).

**Movie S3** Simulation of a generic palmately lobed leaf (corresponds to Fig. 8a–e).

**Movie S4** Simulation of the development of a representative leaf from the 2D morphospace in Fig. 9 (row 3, column 4).

**Movie S5** Simulation of the development of a generic palmate leaf with sequential emergence of lobes (corresponds to Fig. 10a).

**Movie S6** Simulation of the development of a pinnately compound leaf (corresponds to Fig. 12d).
