# Supplementary material for: A common developmental program can produce diverse leaf shapes
Source: New Phytol. 2017 Mar 1;216(2):401–18. doi: 10.1111/nph.14449 (PMC5638099; doi:10.1111/nph.14449)
Supplement: Supplementary file 2 — Table S1 Parameter values used in simulations [file NPH-216-401-s002.pdf]

S1 Parameters used in simulations

				Simulation																																						
**Parameter names**	**Symbol**	**Description**	**Default value**	6a-f	6g	6h	6i	6j	6k	6l	6m	6n	6o	6p	6q	6r	6s	6t	7a	7b	7c	7d	7e	7f	7g	7i	8a-e	8g	8h	8f	8i	9	10a	10b	10c	11	12a	12b	12c	12d/12e	12f	12g
**Initialization**																																										
Primordium dimensions (see note *A* below)		Width		9.9	6.6	---	---	---	---	---	---	---	---	---	9.9	---	---	---	6.6	---	---	---	---	---	---	9.9	8.8	---	---	0.88	8.8	3.52	26.4	---	---	33	26.4	8.8	26.4	17.6	---	---
		Height		13.5	---	---	6.75	---	---	---	---	---	---	---	13.5	---	---	---	---	---	---	---	---	---	---	---	36	---	---	---	---	14.4	108	---	---	136.5	108	54	108	80	---	---
**Margin patterning and morphogens**																																										
Number of morphogens				1	---	---	0	---	---	---	---	---	---	---	1	---	---	---	---	---	---	---	---	---	---	2	---	---	---	---	---	---	3	---	---	---	---	---	---	---	---	---
Sampling threshold		Threshold distance at which new samples are added to the margin.		500	---	---	---	---	---	---	---	---	---	---	---	---	---	---	---	---	---	---	---	---	---	---	3	2	---	3	2	---	50	150	30	40	30	---	150	20	---	---
Boundary distance		Margin distance values at start and end of the margin (symmetric except when two values are specified).		6.25	3.125	---	2.08333	---	---	---	---	---	---	---	4.16667	---	---	---	8.33333	---	---	---	---	---	---	10.3333	3	1	---	2	(-1,-5)	8	2	---	---	20	2	6	2	0	---	---
Axis Boundary	delta	When a CP is added sample points within distance delta are associated with the CP. The boundary between regions associated with CPs may be an interval instead of a point. In this case two values are provided.		1.25	---	---	3.75	---	---	---	---	---	---	---	1.25	---	---	---	---	---	---	---	3.125	---	---	3.875	9	---	---	---	---	---	---	---	---	24	9	5.85	9	(4.05,6)	---	(6.75,9.75)
Fill Sinuses		Limits patterning to sub-apical regions by introducing morphogens into intervals of the margin that do not contain a CP.	FALSE																								TRUE	---	---	---	---	---										
Internal propagation		Permit inward propagation while webbing.	FALSE				TRUE	---	---	---	---	---	---	---													TRUE				TRUE					TRUE						
Margin without morphogen																																										
CP threshold	lambda	Threshold distance for CP introduction.		12.5	---	---	---	---	---	---	---	---	---	---	---	---	---	---	---	---	---	---	---	---	---	15.5	18	---	---	---	---	---	NA	---	30	240	28	32.5	10	NA	---	---
Interval of exclusion of all morphogens	rho_min	Interval where morphogens are eliminated when a CP is introduced.		0.625	---	---	NA	NA	NA	NA	NA	NA	NA	NA	0.625	---	---	---	---	---	---	---	---	---	---	0.775	3	---	---	---	---	---	---	---	---	12	3	1.95	3	0.75	---	---
Distance accumulation rate		Rate at which distances accumulate.		1	---	---	---	---	---	---	---	---	---	---	---	---	---	---	---	---	---	---	---	---	---	---	0.75	---	---	---	---	---	NA	---	0.15	1	0.15	---	1	NA	---	---
Spatial Competence		Region of morphogenetic competence (measured from the base along veins).	∞																														NA	---						NA	---	---
Temporal Competence		Temporal window of morphogenetic competence.	∞																														NA	---						NA	---	---
Patternable		Whether CPs can form. When FALSE margin distances in this region are zero.	TRUE																														FALSE	---		FALSE			FALSE	---	---	---
Stretch	alpha_s	Rate of webbing due to stretching minimization.		0.01	0.02	---	0.5	---	---	---	---	---	---	---	0.01	---	---	---	0.41	0.01	---	0.025	0.00375	---	---	0.0225	0.1375	0.7625	---	0.1	0.07625	1.14375 (row 1), 0.7625 (row 2), 0.38125 (row 3), 0.266875 (row 4), 0.1525 (row 5)	10.5	10.5	0.05	0.0025	0.05	0.3	10.5	1.53	---	---
Curvature 1	alpha_kappa1	Rate of webbing due to minimization of discrete curvature term 1.		0	---	---	0.04	---	---	---	---	---	---	---	0	---	---	---	0.001	0.00025	0	---	0.0000075	---	---	0	---	0.005	0	---	---	---	---	---	---	0	0	---	---	0.1	---	---
Curvature 2	alpha_kappa2	Rate of webbing due minimization of discrete curvature term 2.		0	---	---	0	---	---	---	---	---	---	---	0	---	---	---	0.001	0	---	---	0.000075	---	---	0	---	0.6	---	0	---	0.9 (row 1), 0.6 (row 2), 0.3 (row 3), 0.21 (row 4), 0.12 (row 5)	0.5	---	0	0.00025	0	0.005	0.5	0.05	---	---
Normal	alpha_N	Rate of webbing due to normal propagation.		0	---	---	0	---	---	---	---	---	---	---	0	---	---	---	0.0002	0	---	---	0	---	---	---	5.00E-005	0.000625	0.0003125	0.0000375	3.13E-005	0.000271875 (row 1), 0.00018125 (row 2), 9.0625e-05 (row 3) 6.34375e-05 (row 4), 3.625e-05 (row 5)	0.002	0.0041	0	0	0.00003	0.00006	0.004	3.00E-005	---	---
Morphogen 1																																										
CP threshold	lambda			∞	---	---									∞	---	---	---	---	---	---	---	---	---	---	---	18	---	---	---	---	---	30	42	30	96	30	32.5	70	22.5	---	15
Interval of exclusion for morphogen 1.	rho_min	Interval in which morphogen 1 is eliminated		0.625	---	---									0.625	---	---	---	---	---	---	---	---	---	---	0.775	3	---	---	---	---	---	---	---	---	12	3	1.95	3	0.75	---	---
Interval of introduction	[rho_min,rho_max]	Interval in which morphogen 1 is introduced (if applicable)		NA	---	---									NA	---	---	---	---	---	---	---	---	---	---	---	3.6	---	---	---	---	---	NA	---	---	---	---	---	---	---	---	---
Metric multiplier				0.01	---	---									0.01	---	---	---	---	---	---	---	---	---	---	---	0.75	---	---	---	---	---	0.1	---	0.15	1	0.15	---	0.1	0.25	---	---
Spatial Competence			∞																					---	---	20	42	48	---	27	60	26.4				6000				1000	---	---
Temporal Competence			∞																					---	---						2500											
Patternable				TRUE	---	---									TRUE	---	---	---	---	---	---	---	---	---	---	---	---	---	---	---	---	---	---	---	---	---	---	---	---	---	---	---
Stretch	alpha_s			5.000E-008	---	---									2.500E-008	---	---	---	1.03E-006	2.50E-008	---	6E-008	1.25E-008	---	---	7.50E-008	0.01375	0.07625	---	0.01	0.022875	0.604554 (row 1), 0.330417 (row 2), 0.114375 (row 3), 0.0664348 (row 4), 0.0291136 (row 5)	0.105	1.05	0.005	0.045	0.004	0.3	1.05	0.27	---	---
Curvature 1	alpha_kappa1			0	---	---									0	---	---	---	---	---	---	---	---	---	---	---	---	---	---	---	---	---	---	---	---	0.0015	0	---	---	---	---	---
Curvature 2	alpha_kappa2			0	---	---									0	---	---	---	---	---	---	---	---	---	---	---	---	---	---	---	---	---	0.005	0.05	0	0.0015	0	0.005	0.05	0.25	---	---
Normal	alpha_N			0	---	---									0	---	---	---	---	---	---	---	---	---	---	---	5.00E-006	0.0000625	3.13E-005	2.813E-006	9.38E-006	7.18527e-05 (row 1), 7.85417e-05 (row 2), 2.71875e-05 (row 3), 1.57919e-05 (row 4), 6.92045e-06 (row 5)	1.00E-005	0.0004	0	0	2.40E-006	0.00006	0.0004	0.000125	---	---
Morphogen 2																																										
CP threshold	lambda																										∞	---	---	---	---	---	20	---	---	80	20	13	20	60	---	---
Interval of exclusion for morphogen 2	rho_min																										3.6	---	---	---	---	---	6	---	---	24	6	3.9	6	3.75	1.5	---
Interval of introduction	[rho_min,rho_max]																										NA	---	---	---	---	---	---	---	---	---	---	---	---	---	---	---
Metric multiplier																											0.75	---	---	---	---	---	0.5	---	---	1	0.5	---	---	0.75	---	---
Spatial Competence																											2410.8	---	---	---	---	---	60	---	65	150	65	30.55	60	165	---	---
Temporal Competence																											90000	---	---	---	2500	90000	∞	0 (Simple), 1000 (3 lobes), 2000 (5 lobes), 5000 (7 lobes)	1000 (3 lobes), 3000 (5 lobes), 6000 (7 lobes), 9000 (9 lobes)	∞	7000	---	---	33000	22000	17000
Patternable																											TRUE	---	---	---	---	---	---	---	---	---	---	---	---	---	---	---
Stretch	alpha_s																										0.000275	0.0000275	---	0.000275	2.75E-005	---	0.0021	0.0002625	0.000000625	0.00606323	6.25E-007	0.000225	7.88E-005	0.0002	---	---
Curvature 1	alpha_kappa1																										0	---	---	---	---	---	---	---	---	0	0	---	---	---	---	---
Curvature 2	alpha_kappa2																										0	---	---	---	---	---	---	---	0.000375	0	0.000375	0.0000375	3.00E-008	0	---	---
Normal	alpha_N																										0	---	---	---	---	---	2.00E-007	0.0000001	0	0	3.75E-007	3.75E-008	0	---	---	---
Morphogen 3																																										
CP threshold	lambda																																∞	---	---	---	---	---	---	NA	NA	NA
Interval of exclusion for morphogen 3	rho_min																																9	---	7	36	7	4.55	9	4.5	2.25	6.75
Interval of introduction	[rho_min,rho_max]																																NA	---	---	---	---	---	---	5.25	---	9.75
Metric multiplier																																	1	---	---	---	---	---	---	NA	---	---
Spatial Competence																																	15000	---	---	1500	15000	---	---	NA	---	---
Temporal Competence																																	∞	---	---	---	---	---	---	NA	---	---
Patternable																																	TRUE	---	---	TRUE	TRUE	---	---	FALSE	---	---
Stretch	alpha_s																																2.10E-006	2.625E-007	6.25E-010	0	6.25E-010	0.000000225	6.563E-008	1.00E-006	---	---
Curvature 1	alpha_kappa1																																0	---	---	0	0	---	---	---	---	---
Curvature 2	alpha_kappa2																																0	---	0.000000375	0	3.75E-007	3.75E-008	2.5E-011	0	---	---
Normal	alpha_N																																0	---	---	0	0	---	---	---	---	---
**Growth**																																										
Uniform growth rate			0																				0.0000005	0.000001	0.0000015	2.00E-006					2.00E-006	---										
Webbing																																										
Spatial extent of webbing	Th_webbing	Webbing parameters (alpha_s, alpha_kappa1, alpha_kappa2, alpha_n) decrease linearly to zero at Th_webbing from the leaf base (measured along veins).	∞																									6000	---		60000									2300	---	---
Vein tip																																										
Tip growth rate	Alpha_tip	Rate of vein tip extension in the direction of the vein.		0.00007	0.00007 (mid-vein), 0.000028 (lateral veins)	0.00007 (mid-vein), 0.000014 (lateral veins)	7.00E-008	3.50E-006	7.00E-006	2.10E-005	3.50E-005	4.50E-006	---	9.00E-006	0.00007	---	---	---	---	---	---	---	0.0000375	---	---	9.75E-005	0.00025	0.0005	---	0.000175	0.0001625	0.00025	0.00085	0.0005	0.0003	0.0005	0.0003	0.0001	0.00035	0.0002	---	---
Spatial extent of tip growth	Th_tip	Tip growth decreases linearly from Alpha_tip to zero at Th_tip from the leaf base (measured along veins).	∞									200	75	50														5800	---		58000									2300	---	---
Age dependent growth	Alpha_CP	Rate of increased/decreased tip-growth immediately following CP formation.	0																													-0.000175(Column 1,2), 0(Column 3), 0.000175(Column 4)		0.00175				0.0005	0.003			
Age dependent duration	Th_CP	Duration of increased/decreased tip-growth immediately following CP formation.	0																													640000(Column 1), 80000(Column 2), 0(Column 3), 60000(Column 4)		35000				80000	60000			
Vein elongation																																										
Vein elongation rate	Alpha_vein	Rate of vein elongation.		8.333E-007	---	---	---	---	---	---	---	---	---	---	---	---	---	---	---	---	---	---	3.3333E-007	---	---	---	8.33E-007	2.083E-006	---	8.333E-007	2.08E-006	---	8.33E-007	---	---	1.67E-006	8.33E-007	---	---	1.67E-006	---	---
Spatial extent of vein elongation	Th_vein	Vein elongation decreases linearly from Alpha_vein to zero at Th_vein from the leaf base (measured along veins).	∞				200	---	---	---	---	100	37.5	25														5700	---		57000					4000				1500	---	---
**Vascular patterning**																																										
Resistance ratio	(v/b)	Resistance in veins (v) over that of the blade (b).		0.904827	0.876307	---	0.707107	---	---	---	---	---	---	---	0.961339	0.728969	0.961339	---	0.876307	---	---	---	0.891007	---	---	0.728969	0.865501	---	---	---	---	---	---	---	---	0.809017	0.865501	---	---	---	---	---
Branching angle (radians)	Theta	Corresponding branching angle for v/b (radians).		0.14	0.16	---	0.25	---	---	---	---	---	---	---	0.0888	0.24	0.0888	---	0.16	---	---	---	0.15	---	---	0.24	0.167	---	---	---	---	---	---	---	---	0.2	0.167	---	---	---	---	---
Max margin angle (radians)	sigma_max	Maximum angle between forming vein and margin (radians).		0.5	0.275	---	0.2	---	---	---	---	0.3	---	---	0.455	---	0.2975	0.2075	0.275	---	---	---	0.3	---	---	0.15	0.25	---	---	---	0.2	0.25	---	0.24	---	0.2	0.24	0.25	---	0.2	---	---
Local vs global resistance		Local: attach along the shortest path with the given branching angle (adjusted to respect sigma_max). Global: attach to the point minimizing resistance to the leaf base (as constrained by sigma_max).	Global	Local											Local	---	---	---		---	---	---	Local	---	---		Local	---	---	---	---	---										
**Additional information**																																										
Relevant notes							*B*	*B*	*B*	*B*	*B*	*B*	*B*	*B*								*D*	*C*	*C*	*C*	*C, F*					*C*	*C,H*	*E*	*E*	*E*	*G*	*E*	*E*	*E*	*E*		

A: By default, simulations are initialized with 5 regularly spaced points along the left and right side of the primordium (with one common point at the leaf apex). The simulations in Figs. 11, 12d-g use 7 points.

B: In Fig. 6i-p, webbing due to curvature and bending is reduced at the leaf tip to generate a sharp protrusion.

C: Lateral growth due to uniform growth is eliminated from the petioles in Fig. 7e,f,g,i 8i and 9.

D: The asymmetric serrations in Fig. 7d result from increasing webbing by a factor of 15 proximally and decreasing it by a factor of 0.175 distally. No other simulations employ this effect..

E: Visualization of morphogen 1 is suppressed in Fig. 10a-c, 12a-d to make the role of morphogens patterning lobes and leaflets more apparent.

F: In Fig. 7i, to simulate anisotropic growth during secondary morphogenesis the simulation result was scaled non-uniformly.

G: Figure was generated using an older version, parameters given are the best approximation in the unified implementation available at www.algorithmicbotany.org/papers/leaves2017.html.

H: For consistency growth of the leaf tip is also increased/decreased, but for half the duration of lateral Cps

Table S1: Parameter names, descriptions and values for all simulations presented in the main-text. Filled cells indicate values different from the provided default value; "---" indicates the simulation uses the same value as the preceding column. Blue cells indicate the absence of a morphogen from a simulation.
