# Supplementary material for: A common developmental program can produce diverse leaf shapes
Source: New Phytol. 2017 Mar 1;216(2):401–18. doi: 10.1111/nph.14449 (PMC5638099; doi:10.1111/nph.14449)
Supplement: Supplementary file 1 — Fig. S1 Selected terms pertinent to leaf morphology. Notes S1 Image sources and credits. Notes S2 Additional details regarding the implementation of the generative model of leaf form development. Notes S3 A proof of the relationship between resistance and branching angle given in the main text. [file NPH-216-401-s001.pdf]

New Phytologist Supporting Information for the Article
A common developmental program can produce diverse leaf shapes
Figure S1 and Notes S1–S3

Adam Runions[1,2,*], Miltos Tsiantis[2], Przemyslaw Prusinkiewicz[1*]
[1]University of Calgary, Alberta, Canada
[2]Max Planck Institute for Plant Breeding Research, Cologne, Germany

Article acceptance date: December 6, 2016

List of Supporting Information

The following Supporting Information is available for this article:

Supporting Notes (this file)

Figure S1. Selected terms pertinent to leaf morphology.

Notes S1. Image sources and credits.

Notes S2. Additional details regarding the implementation of the generative model of leaf form development.

Notes S3. A proof of the relation between resistance and branching angle given in the main text.

Movies (In all movies, the simulated leaf is dynamically scaled to maintain approximately constant size when viewed. The scale bar indicates reference length.)

Movie S1. Simulation of the development of a generic simple leaf (corresponds to Fig. 6a-f).

Movie S2. Simulation of the development of a leaf with compound teeth (corresponds to Fig. 7g).

Movie S3. Simulation of a generic palmately lobed leaf (corresponds to Fig. 8a-e).

Movie S4. Simulation of the development of a representative leaf from the 2D morphospace in Fig. 9 (row 3, column 4).

Movie S5. Simulation of the development of a generic palmate leaf with sequential emergence of lobes (corresponds to Fig. 10a).

Movie S6. Simulation of the development of a pinnately compound leaf (corresponds to Fig. 12d).

Table S1 (additional file). Parameter values used in simulations.

The source code and parameter files for the model are available from the authors' web site (`algorithmicbotany.org/papers/leaves2017.html`). Simulations can be executed and visualized using the Virtual Laboratory software environment (`algorithmicbotany.org/virtual_laboratory`).

*Authors for correspondence:
runions@mpipz.mpg.de (Adam Runions)
pwp@ucalgary.ca (Przemyslaw Prusinkiewicz).

Supplementary Figure S1

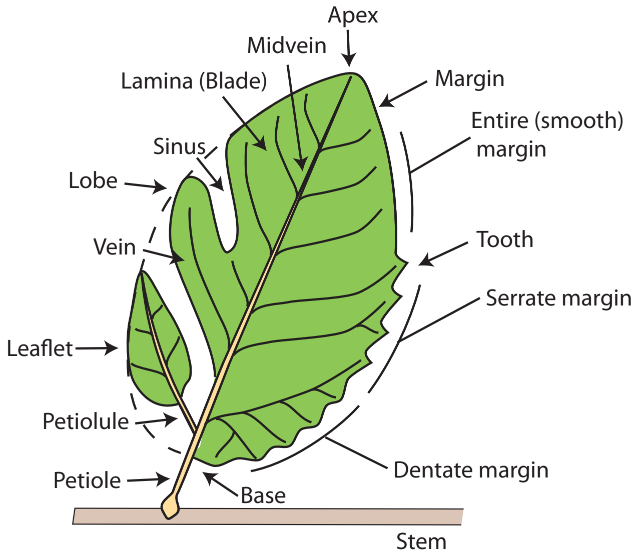

Figure S1: Selected terms pertinent to leaf morphology.

Notes S1. Image sources and credits

Figure 1a. *Quercus imbricaria* by Jan De Langhe, courtesy of Foundation Arboretum Wespelaar (`www.arboretumwespelaar.be`).

Figure 1b. *Garcinia spicata* by Irène Bouguerra and Nicolas Lagarrigue, Pitchandikulam Forest Virtual Herbarium (`www.pitchandikulam-herbarium.org`), licensed under CC-Attribution-NonCommercial-ShareAlike 4.0.

Figure 1c. *Catalpa bignonioides* by W. Mark and J. Reimer, courtesy of SelecTree (`www.selectree.calpoly.edu`).

Figure 1d. *Populus tremula* (`commons.wikimedia.org`), licensed under CC-Attribution-Share Alike 3.0.

Figure 1e. *Crataegus marshallii* by John Pickering, courtesy of Discover Life (`www.discoverlife.org`).

Figure 1f. *Fatsia japonica* (`commons.wikimedia.org`), licensed under CC-Attribution-Share Alike 3.0.

Figure 1h. *Acer saccharinum* by Renn Tumlison, Henderson State University (`www.hsu.edu/Academics/ARNatureTrivia/`), used with permission.

Figure 1i. *Pueraria montana var. lobata* by JK Marlow, courtesy of Native and Naturalized Plants of the Carolinas and Georgia (`www.namethatplant.net`).

Figure 1j. *Cannabis sativa* (`commons.wikimedia.org`), image in public domain.

Figure 1k. *Handroanthus* sp. by Karen Blixen (`www.flickr.com`), licensed under CC-Attribution-Non-Commercial-ShareAlike 2.0.

Figure 1l. *Robinia pseudoacacia* by JK Marlow, courtesy of Native and Naturalized Plants of the Carolinas and Georgia (`www.namethatplant.net`).

Figure 2a. Image from (Hay et al., 2006) adapted with permission from *Development*.

Figure 2b. Image adapted from (Bilsborough et al., 2011).

Figure 2c. Image kindly provided by Gemma Bilsborough.

Figure 7h. *Platanus occidentalis* by Brian Bale (`www.treeplantflowerid.com`), used with permission.

Figure 8f. *Acer macrophyllum* by Dan Anderson (`www.tree-species.blogspot.com`), adapted under fair use.

Figure 8g. *Acer campestre*, photograph from the Middle European Woods dataset (Novotný and Suk, 2013) (`zoi.utia.cas.cz/node/662`), licensed under CC-Attribution-ShareAlike 3.0

Figure 8h. *Acer grandidentatum* by Dean Hueber (`www.pbase.com`), adapted under fair use.

Figure 11. *Morus alba* by Evelyn Fitzgerald (`www.flickr.com`), adapted under fair use.

All remaining images are by the authors.

Notes S2. Model details

S2.1. Maintenance of leaf lamina

The leaf lamina is represented by a trianglular mesh constrained by the leaf margin and the vasculature. It is implemented as a vertex-vertex data structure (Smith et al., 2004).

During simulations the lamina may increase in size by one or two orders of magnitude. This growth is accompanied by significant changes in the form of the leaf. Together, these aspects of the simulation necessitate the use of a dynamic mesh (i.e. a mesh with the number, positions and connectivity of vertices modified over time). Dynamic meshing also permits the faithful approximation of veins and samples on the leaf margin which are dynamically introduced during simulations.

At the beginning of the simulation, the initial mesh is generated as a triangle strip connecting sample points on the two sides of the leaf primordium. During the simulation, the mesh is updated to reflect changes in the margin and the vascular system. The vertices and edges of the mesh that lie on the veins or the margin are moved with the veins and the margin as they grow. Topological changes — the addition of new convergence points or other sample points on the margin, and the insertion of new veins — modify the mesh as shown in Figure N1. Mesh quality is ensured by following these changes with incremental remeshing (Botsch et al., 2010), constrained to preserve mesh edges that lie on the leaf margin or on the veins.

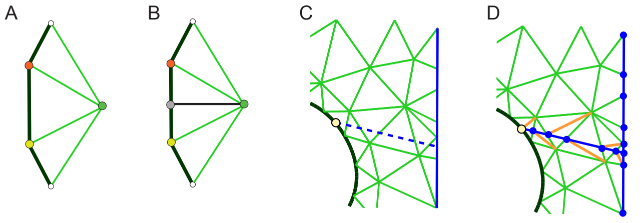

Figure N1: Updating the mesh representing a leaf blade. (A,B) Addition of a new sample point to the margin. The corresponding vertex is added to the mesh, and the incident edge and triangle of the mesh are split. (C,D) Insertion of a new vein (blue line) connecting to an existing vein. As the vein intersects the mesh, the corresponding vertices and edges are added to the mesh. Orange edges split the resulting quads into triangles.

S2.2. Webbing

Let $P_0, P_2, \ldots, P_n$ denote the sequence of sample points defining the margin. To simulate webbing and capture growth directions that do not coincide with veins we iteratively displace each sample point P_i by vector Δ_{W_i} calculated as a weighted sum of four terms:

$$\Delta_{W_i} = \alpha_S \Delta_{S_i} + \alpha_{\kappa 1} \Delta_{\kappa 1_i} + \alpha_{\kappa 2} \Delta_{\kappa 2_i} + \alpha_N N_i. \qquad \text{(S1)}$$

The first three of these terms are geometric fairing terms that smooth the margin by reducing its length and bending. We employ two discrete approximations of curvature to measure bending: the notion of curvature is not uniquely defined in the discrete case, and approximating it using two different terms provides an additional flexibility in controlling details of the margin. The fourth term displaces sample points in the normal direction, thus broadening features of the leaf. The marginal position of vein tips are not modified by webbing as veins are assumed to be rigid compared to other tissues.

To calculate displacements Δ_{S_i}, $\Delta_{\kappa 1_i}$ and $\Delta_{\kappa 2_i}$, we construct energy terms E_S, $E_{\kappa 1}$ and $E_{\kappa 2}$ for the entire margin, and move vertices to reduce the sum of these terms. The stretch energy E_S is calculated as

$$E_S = \sum_{i=1}^{n} \|P_i - P_{i-1}\|^2. \qquad \text{(S2)}$$

The first approximation of curvature at vertex P_i represents it by the turning angle θ_i (Desbrun et al., 2006), yielding

$$E_{\kappa 1} = \sum_{i=1}^{n-1} \theta_i^2. \qquad \text{(S3)}$$

In the second approximation, curvature at vertex P_i is represented by a weighted second finite difference about this point, yielding

$$E_{\kappa 2} = \sum_{i=1}^{n-1} \|w_l^i P_{i-1} - w_c^i P_i + w_r^i P_{i+1}\|^2. \qquad \text{(S4)}$$

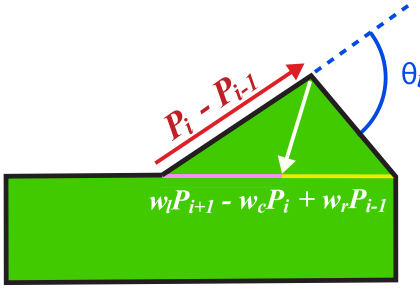

Figure N2: Diagram showing the discrete quantities minimized by webbing. Components of the diagram related to stretching are shown in red, the first curvature approximation κ_1 in blue, and the second curvature approximation κ_2 in white.

We employ the standard second finite difference weights $w_l^i = w_r^i = 1/2$, $w_c^i = 1$, although other weightings are possible (Fornberg, 1988; Desbrun et al., 1999). These weights are chosen so as to produce the vector connecting vertex P_i to the mid-point of the line between P_{i-1} and P_{i+1} (Fig. N2). Webbing is then performed by moving each vertex in the direction that minimizes the above energies, and adding a displacement in the normal direction. As vein tips are not displaced, curvature at these points is minimized by subtracting $\Delta_{\kappa 1_i}/2$ and $\Delta_{\kappa 2_i}/2$ from the corresponding terms at adjacent points prior to performing this displacement.

The coefficients α_S, $\alpha_{\kappa 1}$, $\alpha_{\kappa 2}$ and α_N in Equation S1 depend on the morphogens present at P_i and control the relative contribution of the corresponding four factors to webbing. As these coefficients control growth of the leaf blade (via webbing), they also depend on the distance from the leaf base (as measured along the veins) in the same manner as the growth of the vascular system (see *Growth of the vascular system* in the main text). Furthermore, the displacement in the normal direction is proportional to the average length of the margin edges incident to P_i:

$$\frac{1}{2}\left(\|P_{i+1} - P_i\| + \|P_i - P_{i-1}\|\right). \tag{S5}$$

This displacement thus approximates the effect of an internal pressure acting on the leaf boundary.

S2.3. Visualization of vasculature and the leaf blade

To facilitate visual comparison of real and simulated forms we employed specialized techniques that enhance the visual representation of the models. Visualization of simulation results requires the determination of vein widths and colors for the leaf blade. The width of veins is determined using Murray's law (Murray 1926, see also Runions et al. 2005). Specifically, we use the extension of this law proposed by MacDonald (1983), which relates the radii of veins before and after branching as follows

$$r^n_{parent} = \sum_{i=0}^{k} r^n_{child_i}, \tag{S6}$$

where r_{parent} is the radius of the parent, r_{child_i} are the radii of child veins, and the power n is a species-specific parameter. The applicability of this law to leaf venation was initally supported by tests in sunflower leaves (Roth-Nebelsick et al., 2001). More recently, this law was shown to broadly apply to the vascular patterns of leaves (Price et al., 2014). Following (Runions et al., 2005), vein widths are also increased along the length of veins (moving towards the leaf base), to account for higher-order veins that are not explicitly represented. In Figures 7i, 8f-i, 11 and 12e-g, leaf texture was simulated by specifying a color for vertices coinciding with veins and then stochastically assigning different shades of green to the remaining vertices of the mesh. These colors were then blended to produce continuous changes in the color of the blade.

Notes S3. Branching angle and resistance

In the main text we claimed that introducing a vein so as to minimize resistance to transport causes it to meet an existing vein at a constant branching angle or attach to a branching point. A proof of this claim is given below.

Let b and v be the resistances to transport per unit distance in the leaf blade and vasculature, respectively ($b \geq v \geq 0$). The total resistance to transport from the convergence point to leaf base is then

$$\tau = bA + vB, \tag{S7}$$

where A is the length of the inserted vein, and B is the length of the path from the attachment point to the leaf base (see Figure 5e in the main text). As the length A uniquely determines θ we proceed by finding A minimizing τ. Note that $D \leq A \leq F$, where D and F are the respective distances from the CP to the pre-existing vein, and closest branching point towards the leaf base (or the leaf base if no such branching point exists).

Then by Pythagoras' theorem we have

$$\tau = bA + v(E - \sqrt{A^2 - D^2}), \tag{S8}$$

where $E = C + B$. The length A minimizing τ must satisfy $\frac{d\tau}{dA} = 0$. From this we obtain

$$\frac{d\tau}{dA} = b - vA/\sqrt{A^2 - D^2} = 0. \tag{S9}$$

Then, as $C = \sqrt{A^2 - D^2}$, we have

$$\frac{C}{A} = \frac{v}{b}, \tag{S10}$$

or $\cos(\theta) = \frac{v}{b}$. Thus

$$\theta = \arccos(\frac{v}{b}). \tag{S11}$$

Consequently, the vein minimizing τ meets the existing vein at a constant branching angle $\theta = \arccos(\frac{v}{b})$ or at a branching point (when θ causes A to exceed F).

References

G Bilsborough, A Runions, M Barkoulas, H Jenkins, A Hasson, C Galinha, P Laufs, A Hay, P Prusinkiewicz, and M Tsiantis. Model for the regulation of *Arabidopsis thaliana* leaf margin development. *Proceedings of the National Academy of Sciences*, 108:3424–3429, 2011.

M Botsch, L Kobbelt, M Pauly, P Alliez, and B Lévy. *Polygon Mesh Processing.* A K Peters, Natick, Massachusetts, 2010.

M Desbrun, M Meyer, P Schröder, and A Barr. Implicit fairing of irregular meshes using diffusion and curvature flow. In *Proceedings of the 26th Annual Conference on Computer Graphics and Interactive Techniques*, SIGGRAPH '99, pages 317–324, 1999.

M Desbrun, E Kanso, and Y Tong. Discrete differential forms for computational modeling. In *ACM SIGGRAPH 2006 Courses*, SIGGRAPH '06, pages 39–54, New York, NY, USA, 2006. ACM.

B Fornberg. Generation of finite difference formulas on arbitrarily spaced grids. *Mathematics of Computation*, 51: 699–706, 1988.

A Hay, M Barkoulas, and M Tsiantis. ASYMMETRIC LEAVES1 and auxin activities converge to repress *BREVIPEDICELLUS* expression and promote leaf development in *Arabidopsis*. *Development*, 133(20):3955–3961, 2006.

N MacDonald. *Trees and Networks in Biological Models*. J. Wiley and Sons, New York, 1983.

C Murray. The physiological principle of minimum work I. The vascular system and the cost of blood volume. *Proceedings of the National Academy of Sciences*, 12(3):207–214, 1926.

P Novotný and T Suk. Leaf recognition of woody species in Central Europe. *Biosystems Engineering*, 115(4):444–452, 2013.

C Price, S Knox, and T Brodribb. The influence of branch order on optimal leaf vein geometries: Murray's law and area preserving branching. *PLoS ONE*, 8(12), 12 2014.

A Roth-Nebelsick, D Uhl, V Mosbrugger, and H Kerp. Evolution and function of leaf venation architecture: a review. *Annals of Botany*, 87(5):553–566, 2001.

A Runions, M Fuhrer, B Lane, P Federl, A-G Rolland-Lagan, and P Prusinkiewicz. Modeling and visualization of leaf venation patterns. *ACM Transactions on Graphics*, 24:702–711, 2005.

C Smith, P Prusinkiewicz, and F Samavati. Local specification of surface subdivision algorithms. In J Pfaltz, M Nagl, and B Böhlen, editors, *Applications of Graph Transformations with Industrial Relevance*, volume 3062 of *Lecture Notes in Computer Science*, pages 313–327. Springer, Berlin, 2004.
